# Endogenous nitric oxide promotes *Staphylococcus aureus* virulence by activating autophagy

**DOI:** 10.1128/mbio.04006-24

**Published:** 2025-02-25

**Authors:** Nadira Nurxat, Qichen Wang, Na Zhao, Yanan Guo, Xilong Zhang, Yanan Wang, Ying Jian, Hua Wang, Shengbing Yang, Min Li, Qian Liu

**Affiliations:** 1 Department of Laboratory Medicine, Shanghai Jiao Tong University 12474 , Shanghai, China; 2 College of Health Science and Technology, Shanghai Jiao Tong University School of Medicine 56694 , Shanghai, China; 3 Shanghai Key Laboratory of Orthopaedic Implants, Department of Orthopaedic Surgery, Shanghai Ninth People’s Hospital, Shanghai Jiao Tong University School of Medicine 56694 , Shanghai, China; Cornell University, Ithaca, New York, USA

**Keywords:** nitric oxide, metabolism, *Staphylococcus aureus*, virulence, autophagy

## Abstract

Endogenous nitric oxide (NO) is a small molecule that has been demonstrated to affect the physiology and survival of bacteria. The role of endogenous NO for *Staphylococcus aureus* survival inside host cells remains unclear. Here, we show that the production of endogenous NO by bacterial nitrate reductase (NR) is affected by molybdopterin biosynthesis protein A (MoeA), which is essential for molybdenum cofactor synthesis in *S. aureus*. During the infection, the production of endogenous NO promotes *S. aureus* survival inside macrophages by initiating cellular autophagy. Mechanistically, bacterial endogenous NO can modify the host regulatory protein thioredoxin vis S-nitrosylation, subsequently triggering the phosphorylation of the JNK-Bcl-2 pathway and promoting the initiation of autophagy through the release of Beclin1. Moreover, we confirmed the critical role of MoeA in bacterial survival *in vivo* by using bloodstream infection, pneumonia, and skin abscess model on both wild-type and autophagy-deficient mice. Interestingly, we observed the significantly increased production of NO and activation of cellular autophagy of sequence type (ST)5 compared with ST239, suggesting that the initiation of autophagy is involved in the clone shift of *S. aureus*. Our data offered new insights on the role of bacterial endogenous NO in regulating the host signal pathway during infection inside host cells.

**IMPORTANCE:**

Understanding the mechanism underlying *Staphylococcus aureus* pathogenesis is essential for developing innovative strategies for the prevention and treatment of infection. In this study, we underscore the critical role of molybdopterin biosynthesis protein A and nitric oxide (NO) in inducing autophagy during *S. aureus* survival within macrophage and *in vivo* infection. We demonstrate that host regulatory protein can be modified by bacterial metabolites, which may influence cellular processes. Furthermore, our findings indicated that increased endogenous NO production may contribute to the stable prevalence of *S. aureus* ST5 in the healthcare-associated environment. These findings highlight the significance of bacterial metabolism in modulating the host immune system, thereby facilitating *S. aureus* survival and persistence.

## INTRODUCTION

*Staphylococcus aureus* is one of the leading causes of death from bacterial infection in the world (1). It is commonly known that the pathogenicity of *S. aureus* is due to the secretion of an array of virulence factors (2). These virulence factors, which are under the strict control of regulatory system, are crucial for bacterial survival inside host cells (3).

During the interaction with host cells, the metabolic factors are also crucial for bacterial adaptation to various microenvironments within the host (4). There has been a large number of studies focusing on how metabolic molecules moonlight in virulence factors to combat the host immune system (5). For example, the fatty acid kinase A contributes to the pathogenesis of *S. aureus* by acting as a physiological activator for the SaeRS two-component system by maintaining the cellular lipid homeostasis (6). *S. aureus* glycolysis is essential for virulence by resisting the killing of host immune molecule nitric oxide (NO) during phagocytosis (7). *S. aureus* infection promotes its own survival by inducing cellular autophagy either through decreasing intracellular cAMP levels or through the secretion of α-toxin (8).

The metabolites produced by pathogens during the host infection are also crucial for bacterial survival within the host (9). In bacteria, endogenous NO also serves as a defense mechanism against host neutrophils, antimicrobial peptides, oxidative stress, and cell envelop active antibiotics (10, 11). It is well known that bacteria endogenous NO can be produced by nitric oxide synthases (NOS) through catalyzing the arginine in aerobic or microaerobic condition or by nitrate reductase (NR) through the regulation of the nitrate metabolism in anaerobic condition (12–16). In *S. aureus*, NOS not only protects the bacteria from oxidative stress but also contributes to the nasal colonization and antibiotic resistance (17, 18). The increased vancomycin resistance of *S. aureus* is due to the S-nitrosylation (SNO) of bacterial regulator MgrA or WalR by endogenous NO (19). Although NR is reported to play a crucial role in the virulence of *S. aureus* in an Agr-dependent manner (20), the endogenous NO produced by the activation of NR inside host cells remains to be elucidated.

The NR activity depends on the biosynthesis of the molybdenum cofactor (MoCo), which is synthesized by a series of catalyzed reactions (21). A highly conserved gene cluster, widely distributed in bacteria, is involved in the catalyzed process (22). Among them, molybdopterin biosynthesis protein A (MoeA) catalyzes the conjugation of molybdate to molybdopterin, the last step required for synthesizing bacterial NR activity (21). Previously, we have shown that although MoeA suppressed the activity of the SaeRS two-component system, the deletion mutant of *moeA* still displayed reduced virulence in *S. aureus* (23). It remains unclear how the physiological role of MoeA contributes to the virulence of *S. aureus*.

In this work, we clarified the role of MoeA in the nitrate metabolism in *S. aureus*. The absence of *moeA* displayed significantly impaired NR activity and lower production of bacterial endogenous NO. By using macrophage and mouse infection models, we concluded that the endogenous NO promotes bacterial survival by activating host cellular autophagy during bacterial infection.

## RESULTS

### MoeA affects the transcription of genes involved in nitrate metabolism in *S. aureus*

**Fig 1:**
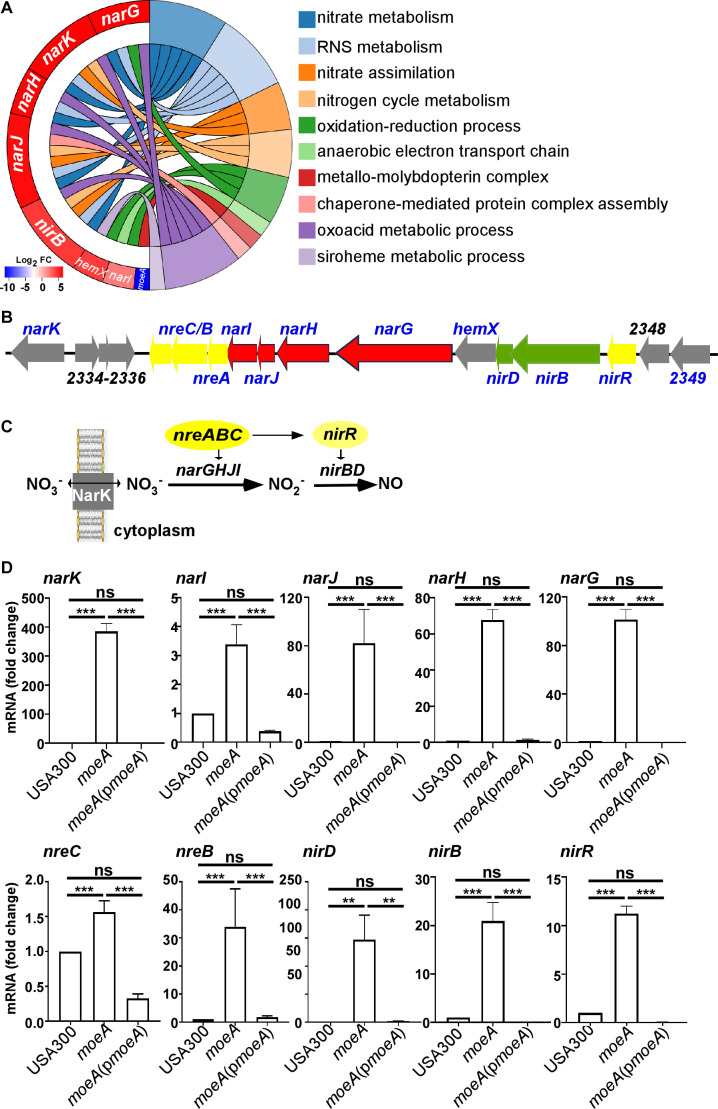
MoeA affects the global transcription of genes involved in nitrate metabolism in *S. aureus*. (**A**) Gene Ontology (GO) enrichment chord diagram. The significantly regulated genes were subjected to functional enrichment analysis to identify their potential roles in biological processes, molecular functions, and pathways. On the left, the genes were arranged in the order of log_2_ fold change (FC) from red to blue, representing the expression levels. Seven significantly upregulated genes, along with *moeA* itself, were clearly labeled. On the right, the GO term information linked with related genes via ribbons. (**B**) The gene cluster involved in nitrate metabolism. Genes encoding NRs were highlighted in red, those encoding nitrite reductases in green, and regulatory genes in yellow. The gene names, whose transcription levels were significantly affected by *moeA* deletion, are highlighted in blue. The numbers of the genes with uncharacterized functions were labeled according to the USA300_FPR3757 strain. (**C**) The nitrate metabolism pathway in *S. aureus*. NarK and the NarGHJI are responsible for nitrate transportation and metabolism. NirBD functions as nitrite reductase, while NreABC and NirR serve as regulators of the nitrate metabolic pathway. (**D**) The transcript levels were confirmed by qRT-PCR for *narK*, *narI*, *narJ*, *narH*, *narG*, *nreC*, *nreB*, *nirD*, *nirB*, and *nirR* genes. The statistical significance was measured by the one-way analysis of variance (ANOVA) (**P < 0.01 and ***P < 0.0001; ns, not significant). USA300, *S. aureus* wild type; *moeA*, *moeA* deletion mutant strain in USA300 background; *moeA*(p*moeA*), *moeA* deletion mutant strain complement with pOS1-*moeA*. Data are shown as mean ± SD from three independent experiments.

We have previously shown the virulence-contributing role of MoeA in *S. aureus* using a bloodstream infection model (23). To elucidate the physiological role of MoeA, we examined the global changes in gene expression. The *moeA* deletion mutant had a significant impact on the transcription levels of 19 genes (6 down-regulated and 13 up-regulated ; Table S1 in the supplemental material). The down-regulated genes included *moeA*, a hypothetical protein (SAUSA300_0793), a putative thioredoxin (Trx) (SAUSA300_0795), and secreted protease *sspABC*. Interestingly, all of the 13 up-regulated genes were associated with bacterial nitrate metabolism (Fig. 1A). In the *S. aureus* genome, a gene cluster is present that regulates nitrate metabolism. Key components include nitrate transporter NarK, the regulatory protein NreABC, the NR NarGHIJ, and the nitrite reductase NirBD (Fig. 1B). Studies have shown that nitrate is transported into the cytoplasm via NarK and subsequently reduced to NO through the enzymatic activities of NarGHIJ and NirBD. NirR, which is regulated by the NreABC complex, functions as a transcriptional regulator for the expression of *nirBD* (24, 25) (Fig. 1C). The elevated expression of 10 genes within the cluster was further verified by real-time quantitative reverse transcription-PCR (qRT-PCR). The transcription of *narK*, *narI*, *narJ*, *narH*, *narG*, *nreC*, *nreB*, *nirD*, *nirB*, and *nirR* genes was increased significantly in the *moeA* deletion mutant strain. This upregulation can be reversed in the complement strain (Fig. 1D), suggesting the potential involvement of *moeA* in *S. aureus* nitrate metabolism.

### MoeA regulates nitric oxide production by affecting the nitrate reductase activity of *S. aureus*

**Fig 2:**
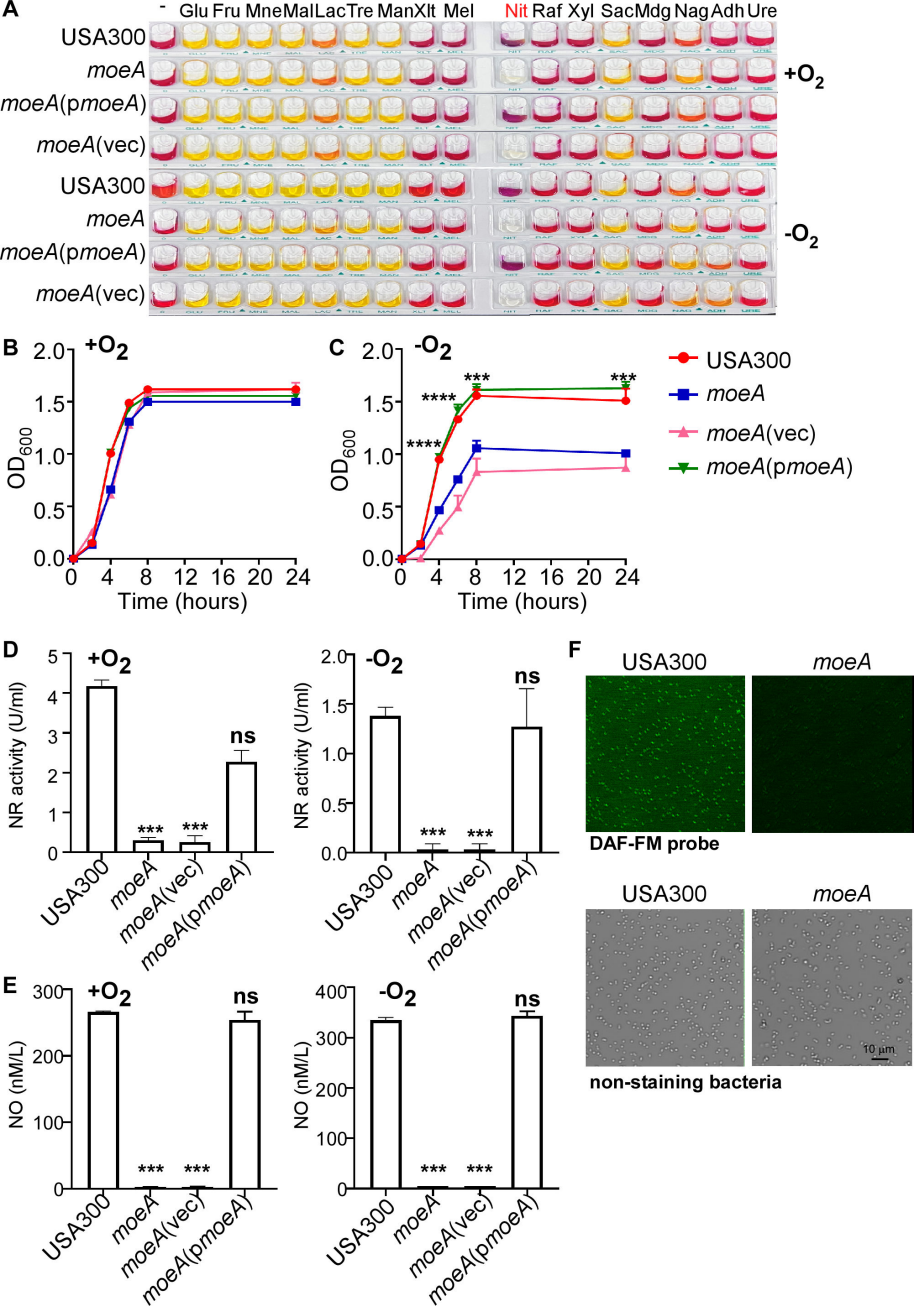
MoeA contributes to endogenous NO production by affecting nitrate metabolism in *S. aureus*. (**A**) The metabolic alterations by *moeA* were screened by API Staph system (20500, Biomerieux, France) under aerobic and anaerobic conditions. The reaction was observed and photographed. Glu, glucose; Fru, fructose; Mne, mannose; Mal, maltose; Lac, lactose; Tre, trehalose; Man, mannitol; Xlt, xylitol; Mel, melibiose; Nit, nitrate; Raf, raffinose; Xyl, xylose; Sac, saccharose; Mdg, Methyl-D-glucopyranoside; Nag, N-acetylglucosamine; Adh, L-Arginine; Ure, urea. (B/C) The growth of different *S. aureus* strains was compared in 2.5% nutrient broth with 0.5% yeast (NY) medium with potassium nitrate (1.67 mM) under aerobic and anaerobic conditions. Statistical significance was measured by linear regression (***P < 0.001 and ****P < 0.0001). (**D**) The effect of *moeA* mutant on NR activity by testing the wavelength at 340 nm. (**E**) The effect of *moeA* mutant on endogenous NO content by NO Content Assay Kit. Statistical significance was measured using one-way ANOVA compared with USA300 (***P < 0.001 and ****P < 0.0001; ns, not significant). (**F**) The NO production was imaged using a Leica TCS SP8 confocal laser scanning microscope. The upper images showed the production of NO labeled by DAF-FM probe, while the lower images showed the bacteria. USA300, *S. aureus* wild type; *moeA*, *moeA* deletion mutant strain in USA300 background; *moeA*(vec), *moeA* deletion mutant strain complement with pOS1 vector; *moeA*(p*moeA*), *moeA* deletion mutant strain complement with pOS1-*moeA*. Data were shown as mean ± SD from three independent experiments.

Based on RNA-sequencing (RNA-seq) data, it is plausible that MoeA participates in bacterial nitrate metabolism, exerting an influence on bacterial growth under anaerobic conditions. By introducing various metabolites under aerobic or anaerobic condition, the ability to convert different metabolites was compared between *S. aureus* USA300 and the *moeA* deletion mutant strains by observing the color changes. We observed that the *moeA* deletion mutant strain exhibited impaired nitrate utilization in contrast to the USA300 strain and this deficiency could be restored by the complement strain (Fig. 2A). A growth defect was observed in the *moeA* deletion mutant strain when nitrate was the sole nitrogen source under anaerobic but not under aerobic condition (Fig. 2B and C). The restored growth of the *moeA* complement strain further confirmed the contribution of *moeA* to nitrate utilization in *S. aureus* (Fig. 2C).

With the validation that MoeA affects the nitrate utilization, we next analyzed the NR activity, which reduces nitrate to nitrite (15). When bacteria were grown in the medium with nitrate as the sole nitrogen source, the *moeA* deletion mutant strain exhibited significantly reduced NR activity. This reduction in NR activity can be restored in the *moeA* complement strain, under both aerobic and anaerobic conditions (Fig. 2D). Additionally, the production of NO, a product of nitrate metabolism, was also significantly decreased in the *moeA* deletion mutant strain (Fig. 2E and F). There were no differences of either NR activity or NO production among different strains when bacteria were grown in the normal tryptic soy broth (TSB) (Fig. S1 in the supplemental material). Taken together, our data suggested that MoeA contributes to *S. aureus* survival by affecting nitrate metabolism.

### MoeA contributes to *S. aureus* survival inside macrophages

**Fig 3:**
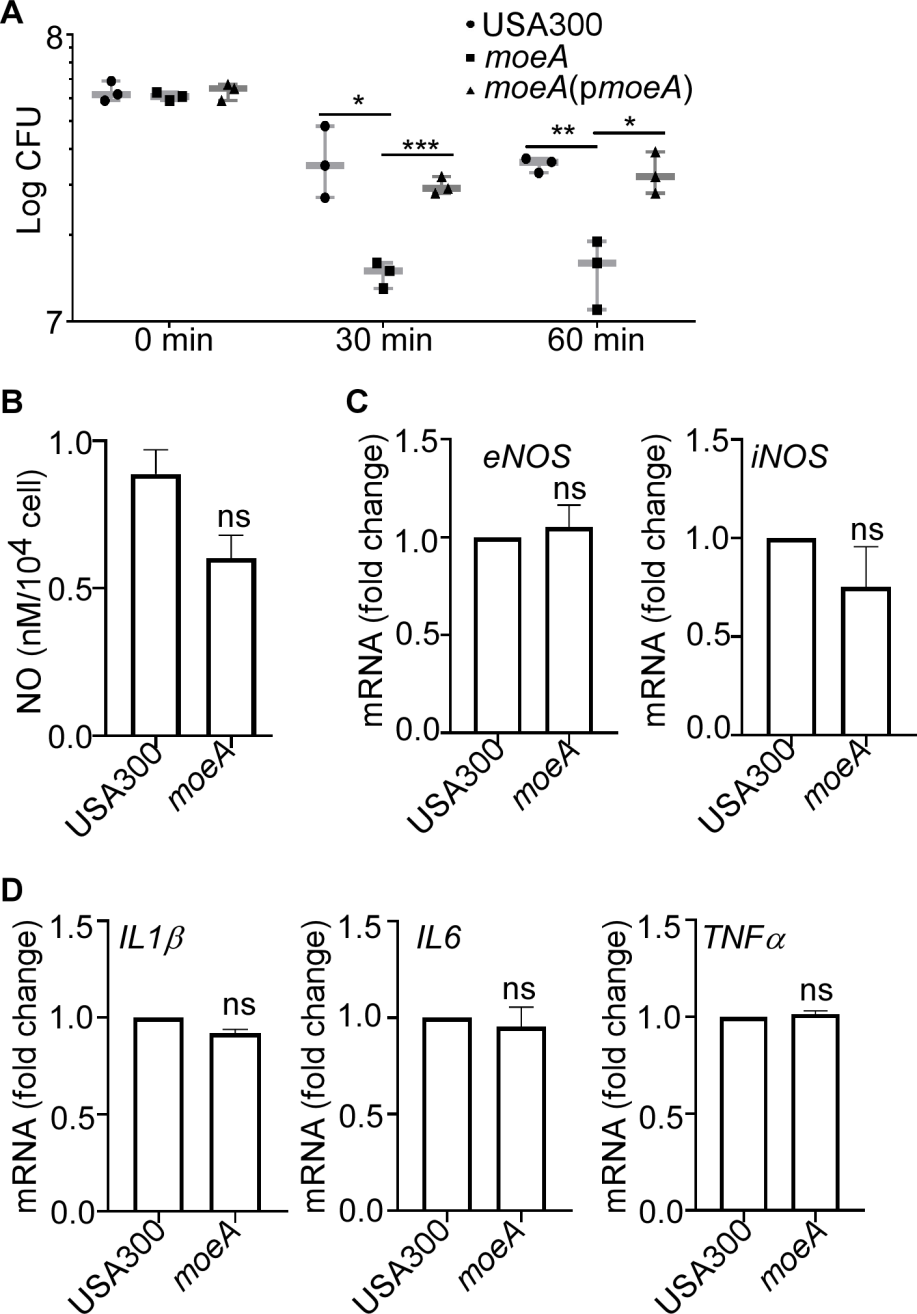
MoeA promotes *S. aureus* survival inside macrophage. (**A**) Survival of *S. aureus* in RAW264.7 macrophages. The bacterial loads were determined at 0 min, 30 min, and 60 min after lysostaphin treatment. The cells were washed and lysed to harvest the viable bacteria inside the host cells. The CFU of bacteria was measured by plating on sheep blood agar plate. The data were collected from three independent experiments. (**B**) The NO contents of the cells were tested by NO Content Assay Kit (Sangon Co.) after 60 min post-infection. The transcription levels of endothelial NOS and inducible NOS (**C**) and *IL1β*, *IL6*, and *TNFα* (**D**) were measured by qRT-PCR after 60 min post-infection. The statistical significance was measured by two-tailed Student’s *t*-test. *P < 0.05, **P < 0.01, and ***P < 0.001; ns, not significant. USA300, *S. aureus* wild type; *moeA*, *moeA* deletion mutant strain in USA300 background; *moeA*(p*moeA*), *moeA* deletion mutant strain complement with pOS1-*moeA*. Data were shown as mean ± SD from three independent experiments.

We next explored whether *moeA* is involved in bacterial survival within host cells. Raw264.7 macrophages were infected with *S. aureus*. After treating with lysostaphin to eliminate extracellular bacteria, we observed a significant decrease in the survival of the *moeA* deletion mutant strain within macrophages compared with the USA300 strain, which could be restored in the complement strain after 30 min and 60 min infection (Fig. 3A).

NO, which serves as a component of the host immune response, can also be produced by macrophages via endothelial NOS (eNOS) or inducible NOS (iNOS) (26). Therefore, we examined the production of NO from host cells after *S. aureus* infection. Interestingly, the NO production and the transcription levels of *eNOS* and *iNOS* from host cells infected with different strains were not affected after 60 min infection (Fig. 3B and C). There were also no differences of the transcription levels of inflammatory factors *IL1β*, *IL6*, and *TNFα* in macrophages infecting with *S. aureus* USA300 and the *moeA* deletion mutant strain (Fig. 3D). Taken together, our data suggested that the contribution of MoeA in defending the killing by host cells is not mainly dependent on inflammation cytokines.

### MoeA plays a role in bacterial survival within macrophage by activating autophagy

**Fig 4:**
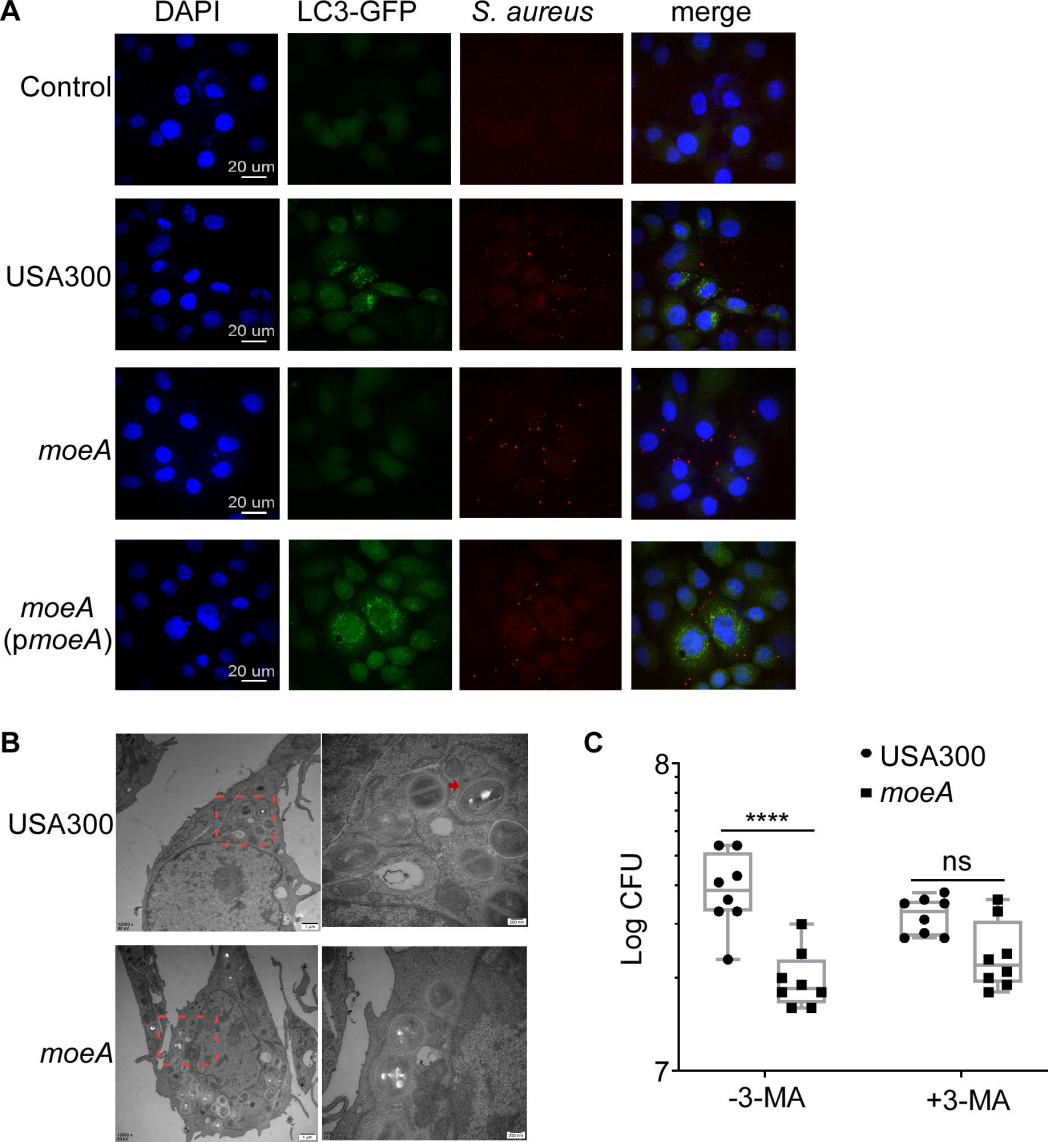
MoeA contributes to bacterial survival in macrophage by activating autophagy. (**A**) Immunostaining images of HeLa cells infected with *S. aureus* (red) and stained with 4',6-Diamidino-2-phenylindole dihydrochloride (DAPI) (blue). LC3, green. Scale bar, 20 µm. (**B**) Electron microscopy analysis of RAW264.7 displayed double-membrane structure enveloping cytoplasmic contents and bacteria, which is autophagosome. The enlargement of the red rectangle part was shown on the right. The typical autophagosome was shown by the red arrow in macrophages infected with *S. aureus* USA300. Scale bar, 1 µm (left) and 200 nm (right). (**C**) Survival of *S. aureus* USA300 and the *moeA* deletion mutant strain in RAW264.7 macrophages treated with 3-MA (3-methyladenine, 2 µM). The data were pooled from two independent experiments. The statistical significance was measured by two-tailed Student’s *t*-test. ****P < 0.0001; ns, not significant. USA300, *S. aureus* wild type; *moeA*, *moeA* deletion mutant strain in USA300 background; *moeA*(p*moeA*), *moeA* deletion mutant strain complement with pOS1-*moeA*.

Autophagy can be induced by *S. aureus* and is essential for bacterial replication at the early stage of infection by inhibiting the fusion of autophagosomes with lysosomes (27–29). To observe cellular autophagy, HeLa cells expressing LC3-GFP were infected with *S. aureus* (30). Cells infected with the *S. aureus* USA300 strain exhibited significantly stronger autophagy compared with those infected with the *moeA* deletion mutant strain, which was restored by the complement strain after 30 min of infection (Fig. 4A). Notably, the typical autophagosomes were also observed in macrophages infected with the *S. aureus* USA300 strain (Fig. 4B).

To determine whether the activation of cellular autophagy by *S. aureus* contributes to bacterial defense against macrophage killing, cells were treated with the autophagy inhibitor 3-methyladenine (3-MA), which inhibits autophagy by targeting the class III phosphatidylinositol 3-kinase (PtdIns3K) complex (31). There were no differences in bacterial loads inside macrophages infected with *S. aureus* USA300 or the *moeA* mutant strain after 3-MA treatment (Fig. 4C). Another inhibitor VPS34, which blocks autophagosome production, also abolished the effect of MoeA on bacterial survival in host cells (Fig. S2 in the supplemental material). In summary, our data suggested that MoeA contributes to *S. aureus* survival by inducing autophagy.

### MoeA-mediated NO production affects autophagy by S-nitrosylation of Trx

**Fig 5:**
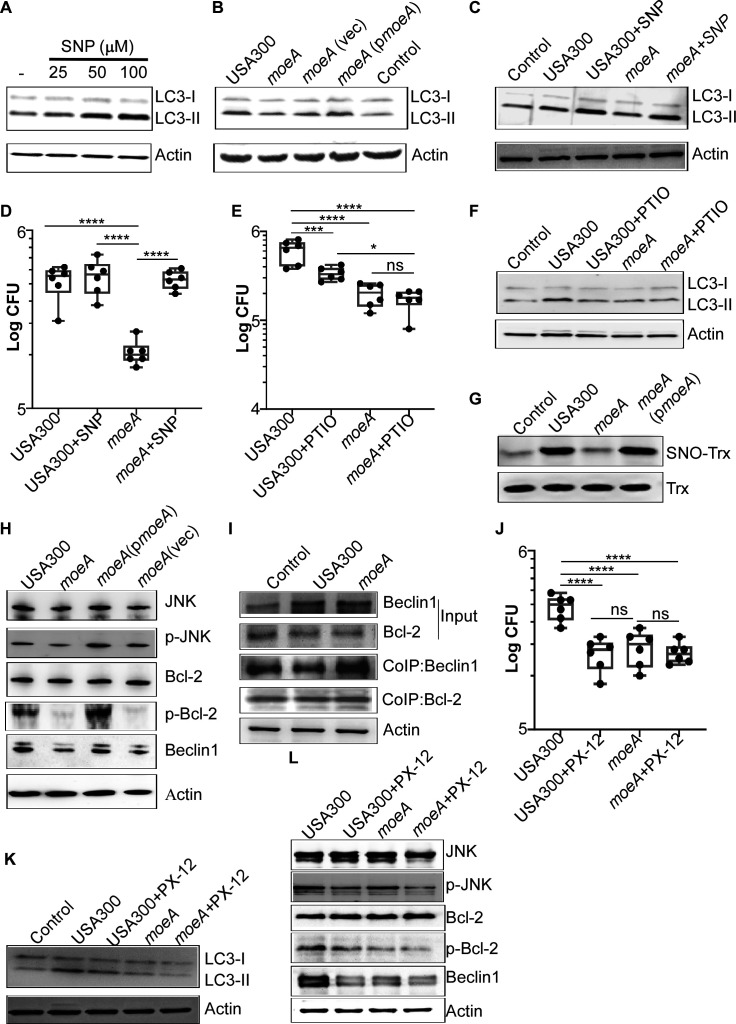
MoeA-mediated NO production affects autophagy by interfering Bcl-2-Beclin1 complex through S-nitrosylation of Trx (SNO-Trx). (**A**) The expression of LC3 was tested in RAW264.7 cells after treatment with different concentrations of SNP for 2 h by western blot. (**B**) Immunoblotting analysis of LC3 in RAW264.7 infected with *S. aureus*. Actin was used as the reference. (**C**) The expression of LC3 was tested in RAW264.7 cells after infecting with *S. aureus* with or without SNP (50 μΜ) for 2 h. (**D**) Survival of *S. aureus* in RAW264.7 macrophages treated with exogenous SNP. After infecting with the USA300 and the *moeA* deletion mutant strains with or without SNP, the bacterial loads were determined by plating on sheep blood agar plate at 60 min after lysostaphin treatment. The data were collected from three independent experiments. (**E**) Survival of *S. aureus* in RAW264.7 macrophages treated with the exogenous carboxy-PTIO (0.5 mΜ). Data were collected from three independent experiments. (**F**) The expression of LC3 was assessed in RAW264.7 cells following infection with *S. aureus*, with or without the addition of carboxy-PTIO (0.5 mΜ) for 2 h. (**G**) The SNO-Trx was tested in RAW264.7 cells by western blot. The cell proteins were collected after infecting with *S. aureus* for 2 h. The S-nitrosylated proteins were labeled, precipitated by TMT antibody and tested using anti-Trx antibody. (**H**) The expression of p-JNK, p-Bcl-2, and Beclin1 levels was tested in RAW264.7 cells infected with *S. aureus*. (**I**) The interaction between Bcl-2 and Beclin1 in RAW264.7 macrophages was tested by co-immunoprecipitation (Co-IP) assay. (**J**) Survival of *S. aureus* in RAW264.7 macrophages treated with exogenous PX-12 (0.5 μΜ). Data were collected from two independent experiments. (**K**) The expression of LC3 was assessed in RAW264.7 cells following infection with *S. aureus* with or without the addition of PX-12 (0.5 μΜ) for 2 h. (**L**) The expression of p-JNK, p-Bcl-2, and Beclin1 levels was tested in RAW264.7 cells infected with *S. aureus* with or without PX-12. Actin was used as the reference. The statistical significance was measured by one-way ANOVA. **P < 0.01, ***P < 0.001, and ****P < 0.0001; ns, not significant. The original gels were shown in Fig. S6. USA300, *S. aureus* wild type; *moeA*, *moeA* deletion mutant strain in USA300 background; *moeA*(vec), *moeA* deletion mutant strain complement with pOS1 vector; *moeA*(p*moeA*), *moeA* deletion mutant strain complement with pOS1-*moeA*. The data are shown based on three independent experiments.

It has been reported that the NO donor Sodium Nitroprusside Dihydrate (SNP) stimulates autophagy in HL-1 and neonatal rat cardiomyocytes (32). We hypothesized that the bacterial endogenous metabolite NO may affect the initiation of autophagy. The different concentrations of SNP were added during the cell culture process, and the conversion from LC3-I to LC3-II was tested by western blot. We observed that the LC3-II content was increased in a dose-dependent manner of SNP (Fig. 5A; Fig. S3A in the supplemental material). The conversion from LC3-I to LC3-II was significantly reduced in macrophages infected with *S. aureus moeA* deletion mutant strain compared with USA300, which can be either reversed in the complement strain or complemented by the NO donor SNP (Fig. 5B and C; Fig. S3B and C in the supplemental material). Meanwhile, the addition of the NO donor also restored the survival of the *moeA* deletion mutant strain inside host cells (Fig. 5D). Interestingly, while SNP promotes the conversion from LC3-I to LC3-II in macrophages infected with both *S. aureus* USA300 and the *moeA* deletion mutant strain, it does not further increase the intracellular bacterial load of the *S. aureus* USA300 strain (Fig. 5C and D). In contrast, in the presence of carboxy-PTIO, an NO scavenger, macrophages infected with *S. aureus* USA300 exhibited significantly lower bacterial loads and reduced conversion from LC3-I to LC3-II (Fig. 5E and F; Fig. S3D in the supplemental material). Collectively, these findings suggested that NO promotes *S. aureus* survival within host cells by activating autophagy.

Then, we aimed to elucidate the specific mechanism of autophagy induction by *S. aureus*. The production of NO may contribute to the hypoxia environment encountered by *S. aureus* within host cells. Hence, we initially examined the expression of hypoxia-inducible factor-1 (HIF-1), a known regulator that promotes autophagy marker BNIP3 in macrophages (33). However, no differences were observed in HIF-1α and BNIP3 levels in the macrophages infected with *S. aureus* USA300 and the *moeA* deletion mutant strain (Fig. S4A in the supplemental material).

The hypoxia environment is not the main reason for the activation of autophagy within the host cells; we turned our attention to investigating another potential pathway. Trx regulates cellular function by performing S-nitrosylation on cysteines (34, 35). The S-nitrosylation of Trx (SNO-Trx) has been shown to promote the release of apoptosis signal-regulating kinase 1 (ASK1), a mitogen-activated protein kinase that subsequently activates c-Jun N-terminal kinase (JNK)-Bcl-2 pathway, thereby promoting the release of free Beclin1 (35–37). Free Beclin1 then plays a crucial role in initiating autophagosome by forming the PtdIns3K complex, leading to the nucleation and assembly of the phagophore membrane structure (37). By comparing the level of SNO-Trx, we observed a significant reduction in SNO-Trx in macrophages infected with the *moeA* mutant strain, which could be restored by the complement strain (Fig. 5G). The decreased phosphorylation of JNK (p-JNK) and Bcl-2 (p-Bcl-2), as well as the lower Beclin1 level and the stronger interaction between Bcl-2 and Beclin1, was observed in macrophages infected with the *moeA* deletion mutant strain compared with the USA300 strain (Fig. 5H1). Notably, there were no differences in the expression of ubiquitin-related proteins including Atg5, Atg7, Atg12, and Atg16L in macrophages infected with *S. aureus* USA300 and the *moeA* deletion mutant strain (Fig. S4B in the supplemental material).

To determine the role of SNO-Trx in the immune evasion of *S. aureus*, 1-methylpropyl 2-imidazolyl disulfide (PX-12), a Trx inhibitor that targets the SNO site Cys73, was introduced in macrophages during bacterial infection (38). Significantly decreased bacterial loads were observed in macrophages infected with *S. aureus* USA300 in the presence of PX-12 (Fig. 5J). Additionally, the conversion from LC3-I to LC3-II was reduced, and the phosphorylation levels of JNK (p-JNK) and Bcl-2 (p-Bcl-2) and the expression of Beclin1 were lower in macrophages infected with the USA300 strain in the presence of exogenous PX-12 (Fig. 5K and L). In summary, our findings suggested that SNO-Trx by NO affects the phosphorylation cascade of JNK-Bcl-2, which ultimately promotes the release of Beclin1 protein and initiates cellular autophagy.

### MoeA contributes to *S. aureus* survival by inducing autophagy *in vivo*

**Fig 6:**
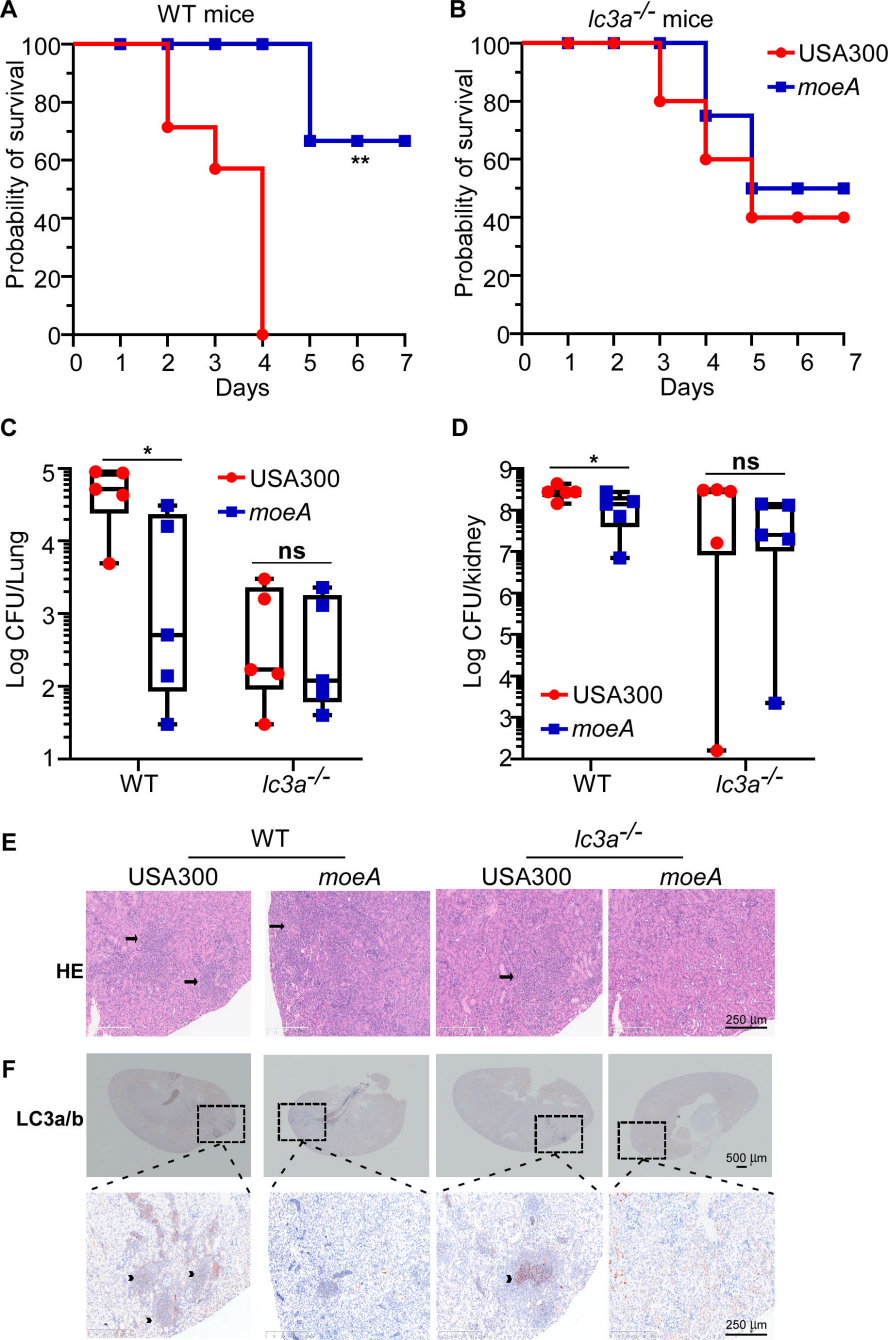
MoeA contributes to *S. aureus* survival by inducing autophagy during host infection. Effect of *moeA* deletion on the mouse survival. (**A**) WT mice and (**B**) autophagy-deficient (*lc3a*^−/−^) mice. The bacteria grown in the exponential growth phase were collected. After washing with phosphate-buffered saline, the bacteria were administered into mice via the retro-orbital injection (*n* = 7). The survival differences were analyzed by log rank (Mantel-Cox) test. The bacterial loads from the lung (**C**) and kidney (**D**) were compared between *S. aureus* USA300 and the *moeA* deletion mutant strain on day 4 post-infection (*n* = 5). The tissues were collected and ground, and the bacterial loads were determined by the serial dilution. Data are shown as mean ± SD. The statistical significance was measured by two-tailed Student’s *t*-test. *P < 0.05; ns, not significant. (**E**) The hematoxylin and eosin (H&E) staining was tested for the kidney section. The arrows showed the inflammation area. (**F**) The activation of autophagy was tested by immunohistochemistry (IHC) using the kidney section. The arrowheads showed the activation of autophagy tested by anti-LC3a/b antibody. WT, the wild-type mice; *lc3a*^−/−^, the autophagy-deficient mice. USA300, *S. aureus* wild type; *moeA*, *moeA* deletion mutant strain in USA300 background. The animal tests were shown from two biological repeats, and the representative data were shown.

Our observations indicate that MoeA-mediated NO production contributes to bacterial survival inside host cells by activating autophagy (Fig. 4 and 5). To test whether autophagy involves in bacterial virulence *in vivo*, we monitored the survival of wild-type (WT) and autophagy-deficient (*lc3a*^−/−^) mice via retro-orbital infection. The significantly decreased survival of the WT mice was observed after infecting with the *S. aureus* USA300 strain (Fig. 6A). *lc3a*^−/−^ mice displayed the similar survival when infected with different *S. aureus* strains (Fig. 6B).

The bacterial loads from the lung and kidney were also significantly increased in the WT mice infected by the *S. aureus* USA300 strain (Fig. 6C and D). There were no significant differences in the bacterial loads in *lc3a*^−/−^ mice infected with different *S. aureus* strains (Fig. 6C and D). We observed much more severe inflammation in the kidney of the WT mice infected by the *S. aureus* USA300 strain by HE staining (Fig. 6E). Consistently, the activation of autophagy in the kidney was much stronger in the WT mice infected with the *S. aureus* USA300 strain compared with the *moeA* deletion mutant by immunohistochemistry (IHC) using anti-LC3a/b antibody (Fig. 6F). Taken together, our data suggested that autophagy contributes to MoeA-mediated *S. aureus* survival *in vivo.*

### MoeA contributes to *S. aureus* acute infection by inducing autophagy *in vivo*

**Fig 7:**
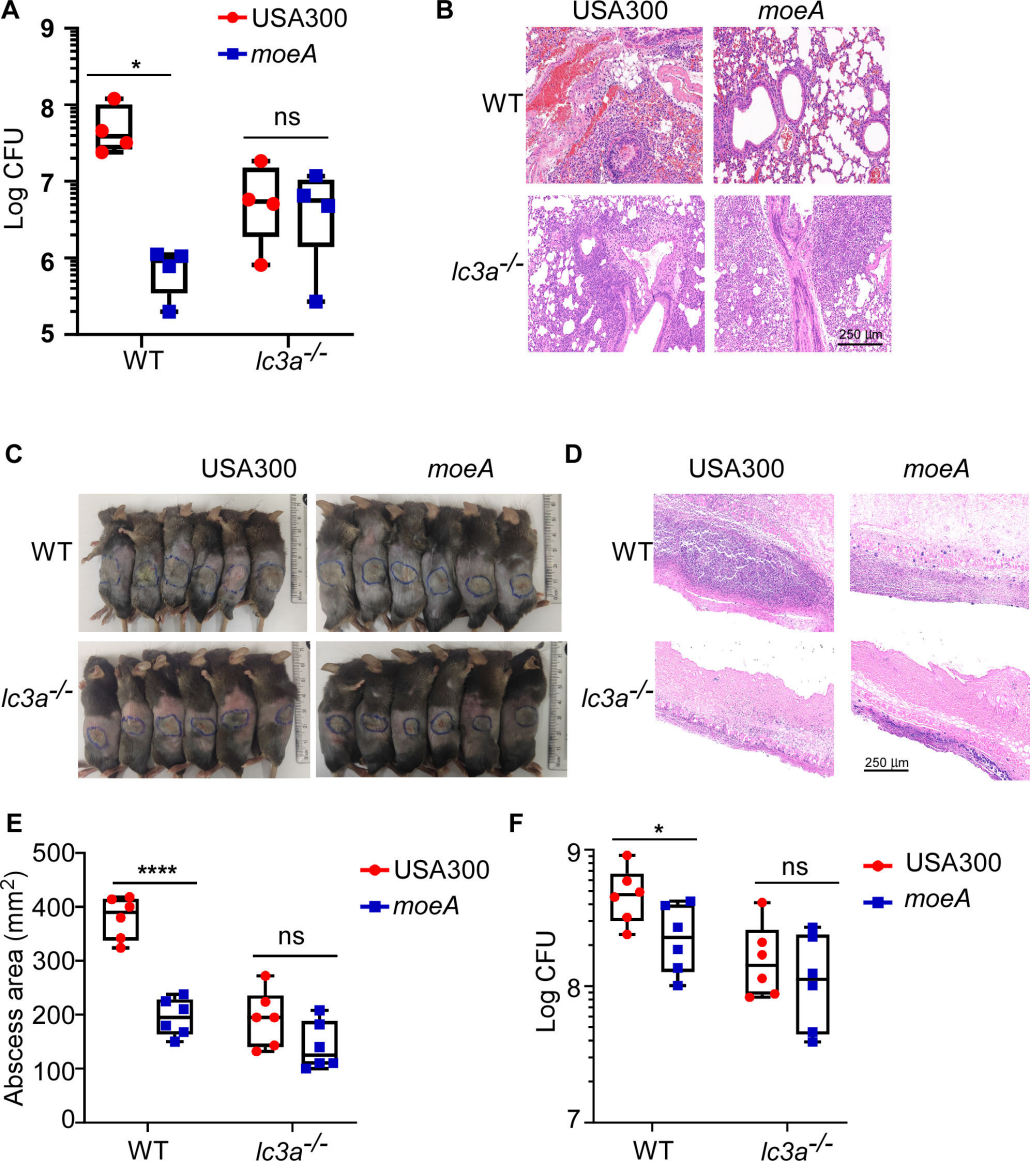
MoeA contributes to bacterial virulence by inducing autophagy *in vivo*. (**A**) The bacterial loads from the lung were compared between *S. aureus* USA300 and the *moeA* deletion mutant strains in acute pneumonia model (*n* = 4). (**B**) The H&E staining was tested for the lung section. (**C**) Photographs of the subcutaneous skin abscesses of mice (*n* = 6) on day 2 post-infection. The H&E staining of the mouse skin tissues (**D**), the abscess area (**E**), and bacterial loads on the skin lesion (**F**) were compared. WT, the wild-type mice; *lc3a*^−/−^, the autophagy-deficient mice. USA300, *S. aureus* wild type; *moeA*, *moeA* deletion mutant strain in USA300 background. Data are shown as mean ± SD. The animal tests were shown from two biological repeats. The statistical significance was measured by two-tailed Student’s *t*-test. *P < 0.05 and ****P < 0.0001; ns, not significant.

It is reported that LC3, a marker for autophagy, rapidly associates with *S. aureus* infection in larval zebrafish, indicating that autophagy is associated with the early stage of infection (39). The role of *moeA* in *S. aureus* was further examined in an acute pneumonia and skin abscess model. After infecting with bacteria for 6 h, the higher bacterial loads, more severe injury, and more infiltration of inflammation cells were observed in the lung of the WT mice infected with the *S. aureus* USA300 strain (Fig. 7A and B). There were no significant differences of bacterial loads or histologic alterations in *lc3a*^−/−^ mice after infecting with different bacterial strains (Fig. 7A and B).

Using the skin abscess model, more obvious skin abscess and stronger local inflammation were also observed in the WT mice infected with the *S. aureus* USA300 strain (Fig. 7C through E). The bacterial loads were significantly increased in WT mice infected with the *S. aureus* USA300 strain (Fig. 7F). These discrepancy disappeared in *lc3a*^−/−^ mice (Fig. 7C through F). Taken together, our data suggested that MoeA contributes to bacterial acute infection by inducing autophagy.

### The induction of autophagy contributes to the clone shift of *S. aureus*

**Fig 8:**
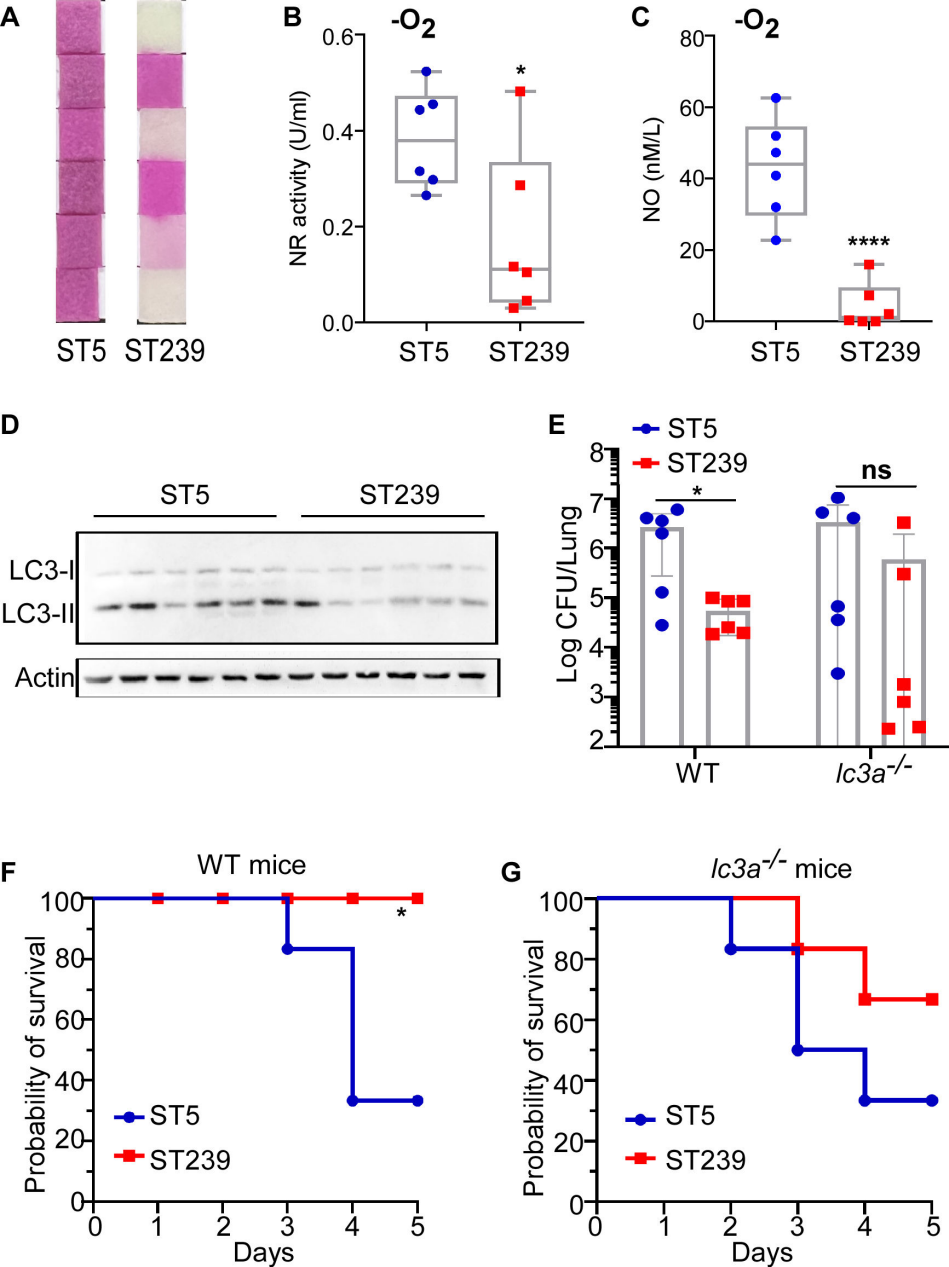
MoeA plays a key role in the stable prevalence of *S. aureus* ST5. (**A**) The utilization of nitrate was compared between ST5 and ST239 under anaerobic condition by API Staph system (20500, Biomerieux, France). The bacteria were added to each reaction well and incubated overnight at 37°C under anaerobic condition. The reaction was observed and photographed. The NR ability (**B**) and NO content (**C**) of *S. aureus* ST5 and ST239 were compared under anaerobic condition. Bacteria were grown in NY liquid medium overnight and normalized by Optical Density (OD_600_). The NR activity in the bacterial pellets was measured by Nitrate Reductase Activity Assay Kit (Sangon Co.), and the NO contents from the cell pellets were measured by NO Content Assay Kit (Sangon Co.). Data were collected from three independent experiments. (**D**) The expression of LC3 was tested in RAW264.7 cells after infecting with *S. aureus* ST5 and ST239. Actin was used as a reference. (**E**) The bacterial loads were compared between *S. aureus* ST5 and ST239 in acute pneumonia model. (**F**) The mouse survival was observed after infecting with *S. aureus* ST5 and ST239. The bacteria grown in the exponential growth phase was collected, washed with PBS, and administered into mice via the retro-orbital injection (*n* = 6, one isolate/one mouse). PBS, phosphate-buffered saline; WT, the wild-type mice; *lc3a*^−/−^, the autophagy deficient mice. The animal test was repeated two times and is shown as mean ± SD from *n* = 6 mice (one isolate/one mouse). The statistical significance was measured by two-tailed Student’s *t*-test. The survival differences were analyzed by log rank (Mantel-Cox) test. *P < 0.05 and ****P < 0.0001; ns, not significant.

*S. aureus* underwent the gradual evolution during the interaction with the host (40, 41). The predominant healthcare-associated *S. aureus* (HA-SA) clone ST239 is replaced by the continually prevalent clone ST5 (42). The changing pattern of the *S. aureus* clone type has been reported to be affected by bacterial growth competition (43). We hypothesized that the ability to utilize nitrate contributes to the bacterial survival inside host cells. Six clinical isolates of ST5 and ST239 obtained from respiratory samples (Table S2 in the supplemental material) were randomly selected for the experiments. We observed that *S. aureus* ST5 strains exhibited a stronger ability to utilize nitrate compared with ST239 (Fig. 8A). Meanwhile, *S. aureus* ST5 strains displayed significantly higher NR activity and NO production compared with ST239 strains when growing in the medium with nitrate as only nitrogen source (Fig. 8B and C).

The gene cluster including *moeA* (Fig. S5A in the supplemental material) is involved in the biosynthesis MoCo, which is required for the activation of NR in bacteria (44). The transcription of the operon was compared between different *S. aureus* strains under the same growth condition. The transcription levels of the *moaA*, *mobB*, *moeA*, *moaB*, and *moeB* genes were significantly higher in *S. aureus* ST5 strains compared with ST239 (Fig. S5B in the supplemental material), suggesting that the higher expression of MoCo biosynthesis genes promotes nitrate utilization.

The endogenous NO contributes to the initiation of autophagy inside host cells (Fig. 5). We observed a significantly higher transition of the autophagy-related protein from LC3-I to LC3-II in macrophages infected with *S. aureus* ST5 compared with ST239 (Fig. 8D; Fig. S5C in the supplemental material), suggesting a stronger induction of autophagy by ST5 strains.

Finally, the pathogenesis of different *S. aureus* strains was compared *in vivo*. The bacterial loads in the lung were significantly higher in WT but not *lc3a*^−/−^ mice infected with ST5 compared with ST239 strains using the mouse acute pneumonia model (Fig. 8E). ST5 strains displayed increased lethality in WT mice but not in *lc3a*^−/−^ mice using the bloodstream infection model (Fig. 8F and G). Taken together, our data suggested that the initiation of autophagy is involved in the clone shift of *S. aureus* by promoting bacterial survival inside host cells.

## DISCUSSION

**Fig 9:**
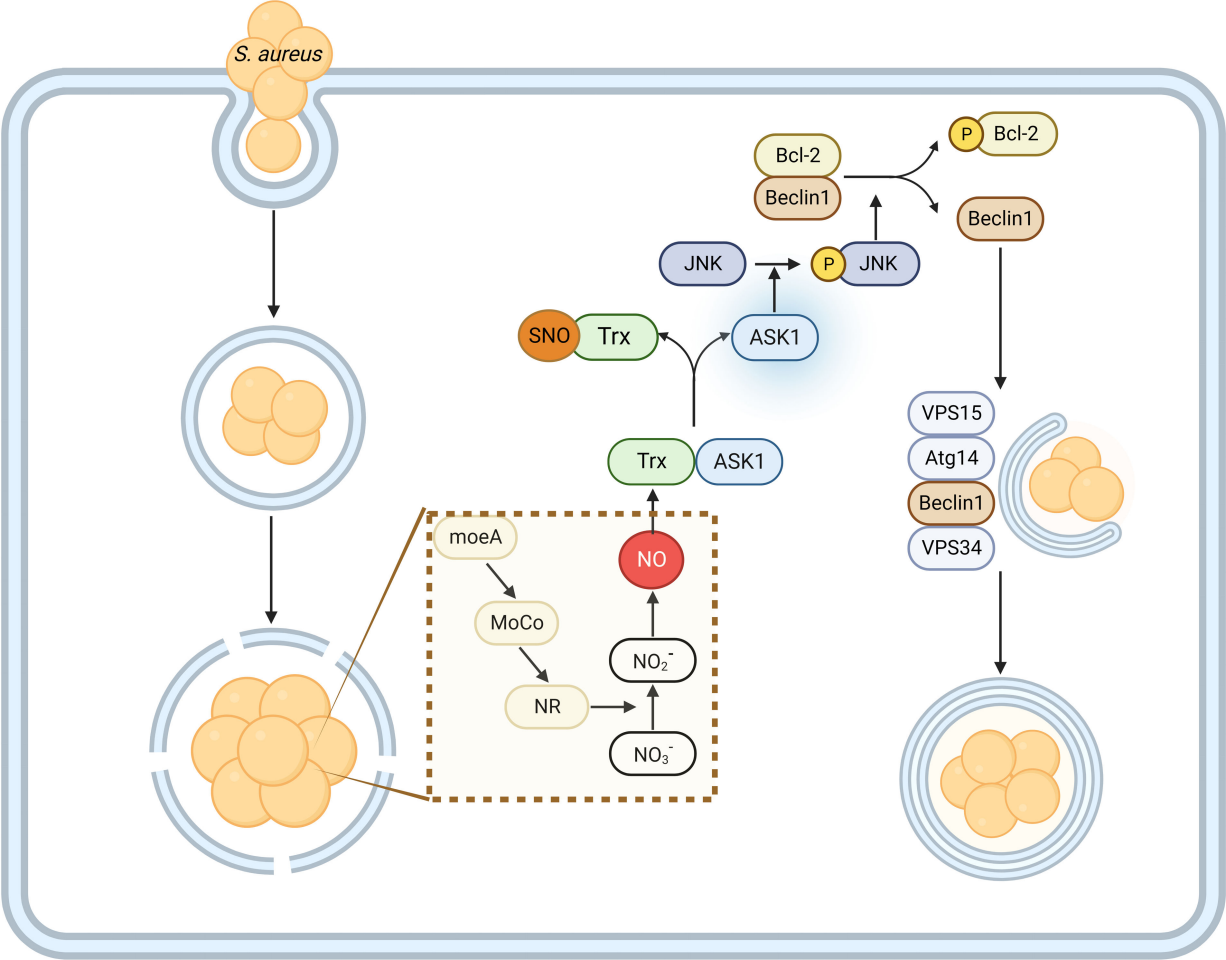
The work model for MoeA-mediated NO production in the pathogenesis of *S. aureus*. Upon infection, *S. aureus* MoeA promotes the synthesis of MoCo, which forms the active site of NR. The activation of NR promotes the intracellular *S. aureus* to utilize nitrate from the host. During the process, the production of bacterial endogenous NO leads to the dissociation of ASK1 by SNO-Trx. The released ASK1 induces phosphorylation of JNK and Bcl-2 sequentially. The phosphorylated Bcl-2 caused the release of Beclin1, an important component for the class III phosphatidylinositol 3-kinase (PI3K), which is crucial for the initiation and the formation of autophagosome. The cellular autophagosome acts as a niche for *S. aureus* to avoid killing from host immune system and replicate. Trx, thioredoxin; ASK1, apoptosis signal-regulating kinase 1; JNK, c-Jun N-terminal kinase; Bcl-2, B-cell lymphoma-2; VPS15, Vacuolar Protein Sorting 15; VPS34, Vacuolar Protein Sorting 34; Atg14, autophagy-related gene 14. VPS15/Atg14/Beclin1/VPS34 form the PtdIns3K.

NO is a small molecule, which plays a diverse role in biological processes and innate immunity in mammalian cells (32). However, the role of bacterial endogenous NO in the pathogenesis of *S. aureus* is still unknown. In the paper, we clarified the physiological role of MoeA in the production of endogenous NO by activating the NR in *S. aureu*s. Critically, the data presented here suggest that the metabolic molecule NO, produced during the nitrate metabolism, is crucial for the bacterial survival in host cells. Mechanistically, the endogenous NO contributes to the initiation of cellular autophagy by modifying the host cell protein Trx, which subsequently increases Beclin1 expression through the phosphorylation of the JNK-Bcl2 signaling cascade (Fig. 9).

MoeA is mainly involved in bacterial nitrate metabolism by testing its role in the utilization of diverse metabolic substrates in *S. aureus* (Fig. 2). The biosynthesis of the MoCo is widely distributed, and the gene cluster involved in the process is also highly conserved in bacteria (21). MoCo is able to form the active site for many metabolic enzymes, which affects bacterial carbon, nitrogen, and sulfur metabolism (14). We hypothesized that the main role of MoCo is diverse depending on the bacteria species. MoCo mainly affects the nitrate metabolism in *S. aureus* in anaerobic condition based on the transcriptional profile and the phenotypes of *moeA* deletion mutant. Consistent with the previous report that there is compensatory elevation due to the absence of the active site associated with *moeA* (45), we also observed the significant elevation in the transcription of genes involved in nitrate metabolism in the *moeA* deletion mutant strain in *S. aureus* (Fig. 1). However, due to the absence of the enzyme’s active site, both NR activity and NO production were significantly reduced in the *moeA* deletion mutant under both anaerobic and aerobic conditions (Fig. 2). Additionally, we observed that the *S. aureus* strain USA300 exhibited higher NR activity under aerobic condition compared with anaerobic conditions. It has been reported that NR synthesis is influenced by culture conditions. NR synthesis reaches its peak during the exponential growth phase in static cultures, whereas it peaks at the onset of the stationary phase in shaking cultures (46). We hypothesized that the higher NR activity under aerobic conditions may be attributed to the timing of sample collection.

In line with the hypothesis that NO produced by nitrate metabolism promotes bacterial replication within host cells, we observed that NO restored the survival of the *moeA* mutant strain by activating cellular autophagy (Fig. 5). Host NO is known as an innate immune molecule defending against pathogens (47). The bacterial endogenous NO also works as defense molecule against killing by the host immune system and antibiotics (10, 11). Although the role of NO in autophagy remains controversial, the NO donor SNP has been shown to stimulate autophagy in HL-1 and neonatal rat cardiomyocytes (32). The role of NO in the initiation of cellular autophagy was elucidated by observing the reduced conversion of LC3-II and the decreased bacterial survival inside macrophages following the addition of the exogenous NO donor SNP or the NO scavenger Carboxy-PTIO (Fig. 5). Mechanistically, NO is involved in the initiation of cellular autophagy through the JNK-Bcl2 phosphorylation cascade by modulating the SNO-Trx in macrophages (Fig. 5). In fact, the exogenous chemicals, including SNP, carboxy-PTIO, or PX-12, significantly increased cytotoxicity in macrophages, even at the lowest concentrations used in the experiment. However, there was no significant difference in the cytotoxic activity of macrophages infected with different *S. aureus* strains when treated with 50 µM SNP, 0.5 mM carboxy-PTIO, or 0.5 µM PX-12 (Fig. S8 in the supplemental material), suggesting that the alteration in bacterial loads is not attributable to the cytotoxicity of macrophages. We also observed that the bacterial loads of *S. aureus* USA300 are influenced by both carboxy-PTIO and PX-12, confirming the role of NO in promoting bacterial replication within host cells. Interestingly, the survival of the *S. aureus* USA300 strain remains higher than that of the *moeA* deletion mutant strain, even when the cells were treated with the NO scavenger carboxy-PTIO (Fig. 5E). We hypothesized that the residual NO produced by *S. aureus* is sufficient to initiate autophagy, which can support the bacterial replication with host cells. We observed that the Trx inhibitor PX-12 significantly reduced the bacterial load of *S. aureus* USA300 to levels comparable to those seen in the *moeA* deletion mutant strain, despite only partially affecting the reversion of LCI to LCII. PX-12 targets the SNO site Cys73 of Trx, suggesting that the nitrosylation of Trx may be only partially inhibited (48). Additionally, PX-12 can induce apoptosis, which may contribute to the killing of intracellular bacteria (49). The exact mechanism on how Trx is modified by NO needs to be further identified.

Consistent with the prior studies, our data indicated the important role of autophagy in the pathogenesis of *S. aureus* (Fig. 6 and 7). Although the main role of autophagy is to maintain homeostasis in eukaryotic cells, certain pathogens promote its own replication inside host cells by hijacking autophagy during the infection (50). The intracellular niche for *S. aureus* in the MAP1LC3 (microtubule-associated protein 1 light chain 3)-associated phagocytosis suggests that autophagy benefits the survival of *S. aureus* (39). Consistent with the epidemiological observation of *S. aureus*, which clarified the dominant clone shift from ST239 to ST5 in the healthcare-associated *S. aureus* (HA-SA) clone in China (42, 51), we confirmed the stronger induction of autophagy by ST5 clone types. Our data suggested that the initiation of autophagy, at least partially, contributes to the stable prevalence of *S. aureus* ST5 in a hospital environment.

Indeed, autophagy plays a crucial role in human innate immunity in defending against pathogens through lysosomal degradation (31, 52, 53). Autophagy inhibits the growth of *Listeria monocytogenes* during the early stage of infection (54). Atg7-regulated autophagy contributes to defense against *Klebsiella pneumoniae* by enhancing bacterial clearance and inflammatory responses (55). Autophagy has also been shown to confer tolerance to *S. aureus* by limiting the damage caused by the important virulence factor alpha-toxin (56). We hypothesized that the multiple factors influence the role of cellular autophagy during infection. Previous studies have demonstrated that autophagy exerts strain-specific effects on *S. aureus*. Strains with high Agr activity can accumulate within autophagosome, thereby creating an intracellular survival niche that protects the bacteria from phagocytic killing (57). Autophagy deficiency affects susceptibility to systemic infection of *S. aureus* USA300 and USA500, but not USA400 (56). We also observed the different induction of autophagy between *S. aureus* ST5 and ST239 clone types (Fig. 8). Nonetheless, these results must be interpreted with caution because we just randomly selected six clinical isolates strains from two clone types. More clone types and strains should be included to confirm the exact role of autophagy in *S. aureus* in our future studies.

In conclusion, we clarified the physiological role of MoeA in the nitrate metabolism by affecting the activity of NR. The bacterial endogenous NO plays a critical role in *S. aureus* survival inside macrophages by initiating cellular autophagy through interfering the cellular regulatory pathway. MoeA is highly conserved in bacteria, suggesting the similar role of MoeA in these organisms. Moreover, our data provide a new sight in understanding the intricate interplay between bacteria, autophagy, and host immune responses, which will potentially lead to the development of novel strategies for combating *S. aureus* infection.

## MATERIALS AND METHODS

### Bacterial strains, plasmids, and culture conditions

The *S. aureus* clinical isolate USA300-0114 without plasmids 2 and 3 was used as the wild type (58). The *moeA* deletion mutant strain and the complement plasmid were constructed in our laboratory in a previous study (23). *Escherichia coli* and *S. aureus* were grown in lysogeny broth and TSB (Oxoid), respectively. The complement plasmid p*moeA* was electroporated into *S. aureus* RN4220 with a gene pulser (Bio-Rad) and then transduced into the *moeA* deletion mutant strain with Φ85.

### RNA-seq and real-time quantitative reverse transcription-PCR (qRT-PCR)

*S. aureus* isolates were grown in TSB with shaking at 200 rpm at 37°C. The overnight cultivated bacteria were diluted at a ratio of 1:100 into fresh TSB and grown for 4 h. Subsequent to centrifugation, the pellets were subjected to mechanical disruption utilizing a FastPrep-96 apparatus (MP Biomedicals Products) at a speed of 800 rpm. Following centrifugation, the supernatant was carefully collected and processed for total RNA isolation (Qiagen).

The Ribo-Zero rRNA 735 Removal Kit (Illumina) was utilized to eliminate rRNA. After the synthesis of complementary DNA (cDNA), 3′ ends of the DNA fragments were adenylated and ligated with Illumina PE adapter oligonucleotides. The libraries were purified using the AMPure XP system (Beckman Coulter) and then selectively enriched using Illumina PCR Primer Cocktail. After purifying by an AMPure XP system, the libraries were sequenced on a NextSeq 500 platform (Illumina) and analyzed using CLC Genomics Workbench 8.0 (Qiagen). Differentially expressed genes were determined by performing a negative binomial test using the DESeq software, with thresholds of *P* value < 0.05 and absolute fold change ≥ 2. The RNA-seq data are available in the Sequence Read Archive (PRJNA988963).

For qRT-PCR, the isolated RNA was then treated with DNase using a TRUBO DNA-free kit (Ambion) to eliminate any potential DNA contamination. A quantity of 1 μg of the purified total RNA was then reverse transcribed into cDNA using a Prime Script RT reagent kit (Qiagen) and subsequent RT-PCR reactions using SYBR (Roche). These reactions were carried out in a MicroAmp optical 96-well reaction plate using a 7500 sequence detector (ABI). The primers are listed in Table S3 in the supplemental material.

### Biochemical identification for measuring the bacterial metabolic level

The bacterial colonies were carefully transferred into the matrix solution supplied by the API Staph kit (Biomerieux), ensuring that the bacterial suspension was adjusted to a 0.5 McFarland standard. Bacterial suspension (100 μL) was subsequently dispensed into each designated reaction well. After incubation for a period of 18 h at 37°C, under both aerobic and anaerobic conditions, the reactions were recorded.

### Nitrate reductase activity

Based on the principle that NR catalyzes the reduction of nitrate (NO3^−^) to nitrite (NO2^−^), the NR activity was detected by testing the absorption wavelength at 340 nm, reflecting the rate of NADH oxidation (Sangon Co. China, D799304). Briefly, bacteria were grown in NY liquid medium (2.5% nutrient broth, 0.5% yeast) overnight and normalized by OD_600_. The cell pellets were resuspended by NR extraction buffer. After sonication, the NR activity was detected on a plate reader (Synergy 2, Biotek).

### Bacteria NO content assay

The bacterial NO contents were either qualified by NO content Assay Kit (Sangon Co.) or visualized by a confocal microscope. Bacteria were grown in NY liquid medium containing potassium nitrate (1.67mmol/L) overnight and normalized by OD_600_.

The cell pellets were resuspended by NO extraction buffer. After sonication, the intracellular NO was qualified by detecting the absorbance value at 550 nm on a plate reader (synergy 2, Biotek).

For the visualization of bacterial NO, the bacterial strains were labelled with DAF-FM (S0019 Beyotime, China) for a duration of 20 min at 37°C. Following this, the bacteria were washed thrice with PBS and subsequently imaged using a Leica TCS SP8 confocal laser scanning microscope (CLCSM).

### Cell culture

*S. aureus* was grown to the exponential growth phase in TSB and washed twice with Roswell Park Memorial Institute (RPMI) 1640. Concurrently, Raw264.7 cell was cultured in RPMI 1640 supplemented with fetal bovine serum (FBS, 10%) with 5% CO_2_ at 37°C. For all macrophage infection models, RAW264.7 cell was infected with *S. aureus* (MOI = 10) for 1 h and then treated with lysostaphin (50 μg/mL) to get rid of the extracellular bacteria. For bacterial CFU counting, the cells were collected at different time points after lysostaphin treatment. The cells were lysed with 0.5% Triton X-100 (500 µL), and the bacterial loads were enumerated by serial dilution on 5% sheep blood agar. For all other experiments, the cells were collected at 1 h after lysostaphin treatment. In some experiments, SNP (an NO donor, abs42027520, Absin, Shanghai, China), carboxy-PTIO (2-(4-carboxyphenyl)-4,4,5,5-tetramethylimidazoline-1-oxyl 3-oxide, an NO scavenger, MX4701, Maokangbio, China), and PX-12 (1-methylpropyl 2-imidazolyl disulfide, an inhibitor of Trx, DB05448, Shelleck) were added.

### Western blot

The protein from macrophage was extracted by RIPA (Radio Immunoprecipitation Assay) lysis buffer (Yeason), and the concentration was measured with a Bicinchoninic acid (BCA) Protein Quantitative Kit (Sangon Co.). After boiling for 10 min, proteins were separated by SDS-PAGE with the assistance of protein loading buffer and transferred to PVDF membranes (Cytiva). In western blot analysis, the following antibodies were used: anti-JNK, anti-p-JNK, anti-Bcl-2, anti-p-Bcl-2, anti-BNIP3, anti-HIF-1, anti-LC3a/b, anti-Beclin1 (rabbit originated, purchased from CST), and anti-Actin (mouse originated, purchased from Yeasen, China),

### Immunofluorescence microscopy

HeLa cell was cultured in Dulbecco’s modified Eagle medium (DMEM) supplemented with FBS (10%) with 5% CO_2_ at 37°C. HeLa cell was infected with *S. aureus* (MOI = 10) for 1 h and then treated with lysostaphin (50 μg/mL) to get rid of the extracellular bacteria. For immunofluorescence assay, the cells were fixed with 4% paraformaldehyde at 1 h after lysostaphin treatment. After fixing for 15 min, the cells were washed with PBS and incubated in PBS containing 0.5% Triton X-100 for 20 min. Upon drying, the slides were blocked with goat serum for 30 min and incubated with anti-SpA antibody (SAB4200745, sigma) at room temperature for 1 h. After washing, the slides were incubated with Alexa Fluor 594 (CST) and then stained by DAPI (Yeason, China). Fluorescence images were captured employing the Leica TCS SP8 CLCSM.

### Transmission electron microscope

After infection with *S. aureus*, the macrophages were fixed with 2.5% glutaraldehyde for 2 h at 4 °C, washed twice by phosphate buffer (PB). After incubating in 1% osmium tetroxide in the PB for 2 h at 4 °C, the cells were rinsed with distilled water twice and subjected to dehydration through a graded ethanol series. The ethanol was then replaced by propylene oxide twice. The samples were subsequently embedded in Epon812, sectioned utilizing a LEICA EM UC7 ultramicrotome, and stained with a 2% lead citrate solution. The cells were observed by TEM (HITACHI, H-7650, Japan).

### Biotin switch assay for detection of S-nitrosylated proteins

The reaction was performed according to the protocol from the Pierce S-Nitrosylation Western Blot Kit (90105, Thermo Scientific). After infection with *S. aureus*, the macrophages were lysed with HENs buffer and centrifuged at 10,000 *g* for 10 min. The proteins in the supernatant were qualified by BCA Protein Quantitative Kit (Sangon Co.). All the samples (1 mg/mL, 100 μL) were mixed with 1M MMTS, incubated at room temperature for 30 min to block free cysteine thiols, precipitated with pre-chilled acetone, and finally resuspended by 100 μL HENs buffer. After vortexing with 1 μL of the labeling reagent, the samples were incubated for 2 h at room temperature for labeling reaction with the assistance of 1M sodium ascorbate (2 μL). The samples were further precipitated by pre-chilled acetone and acetonitrile sequentially. After resolving in 40 mM ammonium bicarbonate and trypsin at 37°C for 4 h, the samples were incubated with TMT antibody and protein A/G Beads (Beyotime, China) overnight at 4°C. After washing with HENs buffer four times gently, the labeled proteins were separated by SDS-PAGE, followed by western blot using rabbit anti-Trx (Abcam).

### Co-immunoprecipitation

After infection with *S. aureus*, the macrophages were treated with RIPA lysis buffer (Yeason), in addition to 1 mM PMSF and phosphatase inhibitors (Absin, China). The proteins were collected by centrifugation at 10,000 *g* for 5 min. The supernatant was incubated with anti-Bcl-2 and protein A/G Beads (Beyotime, China) overnight at 4°C. After washing with lysis buffer four times gently, the co-immunoprecipitated Beclin1 was detected by western blot.

### Mouse model

#### Mouse strains, husbandry, and genotyping

C56BL/6 *map1lc3a*^−/−^ (*lc3a*^−/−^) mice were generated using CRISPR/Cas9 (Shanghai Model Organisms). The homozygous *lc3a*^−/−^ animals were verified by genotyping. Simply, the deletion of the 471 bp fragment on the exon 2–3 of *map1lc3a* gene was confirmed by PCR from tail snips. The PCR product was purified and sequenced using primers listed in Table S2 in the supplemental material. Male and female mice (6–8 weeks old) were housed under specific pathogen-free conditions in filter-top cages that were changed bi-monthly by veterinary or research personnel. Sterile water and food were provided ad libitum. Mouse age, sex, lineage, and source facility were tracked for all experiments.

Bacterial cultures were spun down and washed with sterile PBS before resuspension for infection. Female C57BL/6J mice (6~8 weeks old) were anesthetized with Avertin. Then, a solution of PBS containing bacteria was used for infection.

##### Mouse sepsis model

For the survival curve, the bacterial suspension (around 10^7^ CFU, 100 μL) was administered into the mice (*n*=7 for USA300 and *moeA* mutant strains, *n* = 6 for clinical isolates, 1 mice/isolate) via retro-orbital injection. The infected mice were observed for 7 days. To test the bacterial survival, the bacterial suspension (around 0.5*10^7^ CFU, 100 μL) was administered into the mice (*n* = 5 for USA300 and *moeA* mutant strains) via retro-orbital injection. At day 4 post-infection, all mice were euthanized and organs were harvested. All the tissues were homogenized in 500 μL PBS with several Lysing Matrix Z Bulk beads using an MP homogenizer. Bacterial loads were determined by plating on 5% sheep blood agar.

##### Mouse acute pneumonia model

A solution of PBS containing bacteria (around 10^9^ CFU) was instilled intranasally (20 μL per nostril). After 6 h, mice were euthanized to quantify the bacterial loads in lungs and to observe any pathological alterations in the lung tissue. Lungs were homogenized, and bacterial loads were determined as above.

##### Mouse skin abscess model

The dorsal hair was shaved off to expose the skin, and mice were injected subcutaneously with bacteria (around 10^9^ CFU, 100 μL). After 48 h, mice were sacrificed to measure the area of the skin abscess and to quantify the bacterial burden in the abscess. The skin tissue was cut with an 8 mm diameter punch and homogenized. Bacterial loads were determined as above.

### Hematoxylin and eosin staining and immunohistochemistry

The tissues from the mice were fixed in 4% paraformaldehyde, embedded in paraffin wax, and cut into 5 μm sections. The paraffin sections were deparaffinized and stained with H&E. For IHC, the paraffin sections were incubated with anti-LC3a/b overnight at 4°C. After washing with TBST buffer (20 mM Tris, 150 mM NaCl, 0.1% Tween-20, pH 7.4), the HRP-labeled Goat Anti-Rabbit IgG (Abcam, ab205718) was added and incubated at 37°C for 1 h. The section was observed after adding DAB (Maxim, DAB4033).

#### Cytotoxicity detection

Cytotoxicity was assessed by quantifying the release of lactate dehydrogenase (LDH) following cellular infection. The supernatant from indicated macrophages was collected and analyzed using an LDH cytotoxicity assay kit (Roche) in accordance with the manufacturer’s instructions. The LDH release was normalized to the total LDH content determined in cell lysates treated with 1% Triton-X-100.

#### Statistical analysis

All data were analyzed in Prism 9 software (GraphPad) using two-tailed Student’s *t*-test or one-way ANOVA. ANOVA was employed to compare means among three or more groups, and *post hoc* tests (multiple comparisons) were conducted to analyze the specific pairs. The animal survival was compared by log rank (Mantel-Cox) test with Prism 9 software. *P* < 0.05 was considered as statistically difference.

## SUPPLEMENTAL MATERIAL

Supplemental material mbio.04006-24-s0001.docx Supplemental figures and tables.

ASM does not own the copyrights to Supplemental Material that may be linked to, or accessed through, an article. The authors have granted ASM a non-exclusive, world-wide license to publish the Supplemental Material files. Please contact the corresponding author directly for reuse.
