# Supplementary material for: Endogenous nitric oxide promotes Staphylococcus aureus virulence by activating autophagy
Source: mBio. 2025 Feb 25;16(4):e04006-24. doi: 10.1128/mbio.04006-24 (PMC11980563; doi:10.1128/mbio.04006-24)
Supplement: Supplemental material — Supplemental figures and tables. [file mbio.04006-24-s0001.docx]

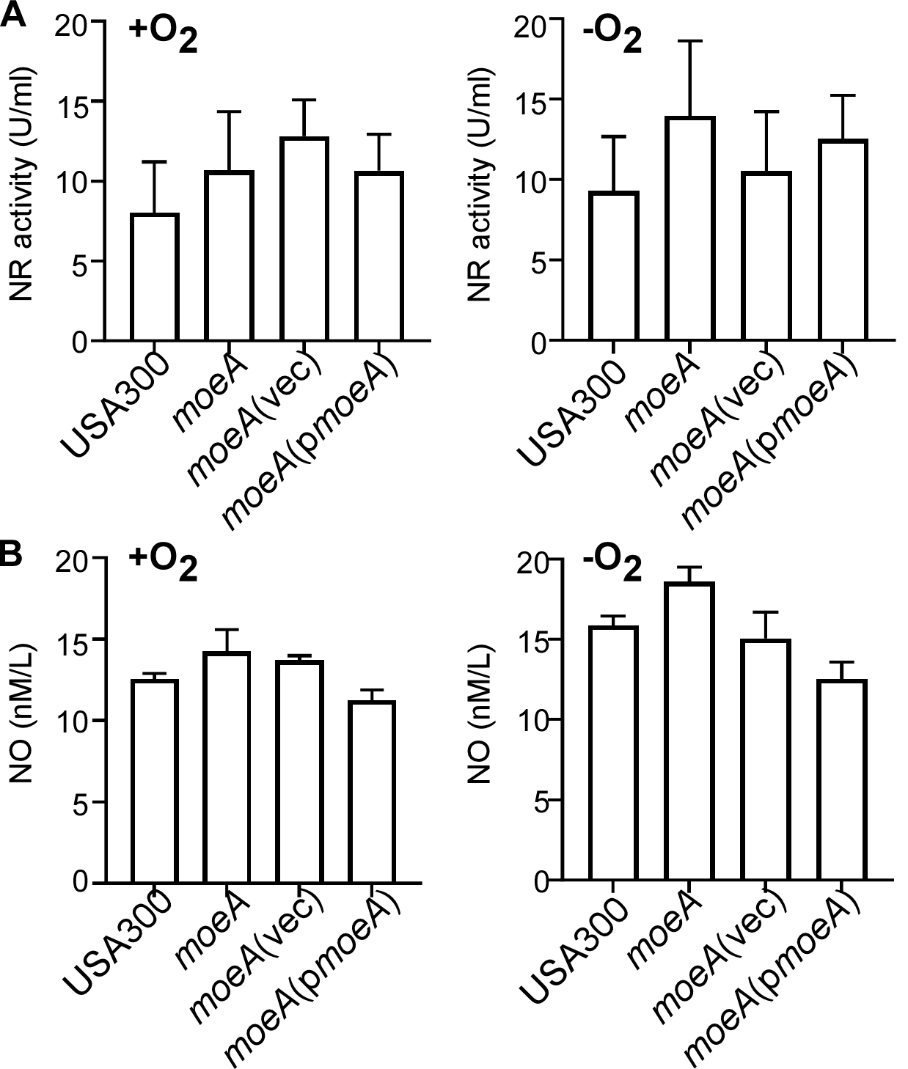


**FIG S1. MoeA did not affect nitrate metabolism in Tryptic Soy Broth (TSB) in *S. aureus*. (A)** The effect of *moeA* mutant on nitrate reductase (NR) activity by testing the wavelength at 340 nm. **(B)** The effect of *moeA* mutant on intracellular NO content by NO Content Assay Kit. *S. aureus* strains were grown in TSB under aerobic and anaerobic conditions. USA300, *S. aureus* wild type; *moeA*, *moeA* deletion mutant strain in USA300 background; *moeA*(vec), *moeA* deletion mutant strain complement with pOS1 vector; *moeA*(p*moeA*), *moeA* deletion mutant strain complement with pOS1-*moeA*. Data were shown as mean ± SD from two independent experiments.


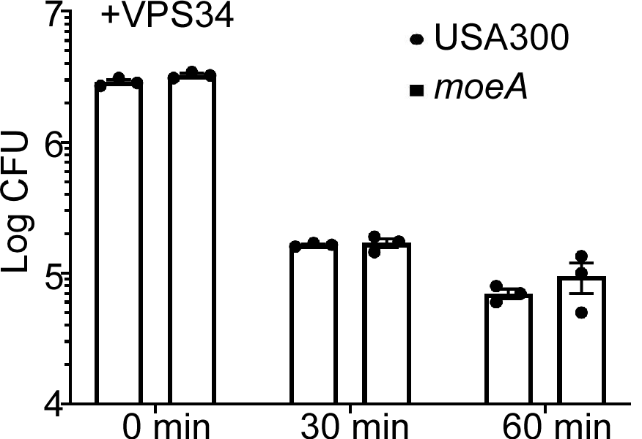


**FIG S2. MoeA contributes to bacterial survival in macrophage by activating autophagy.** Survival of *S. aureus* USA300 and *moeA* mutant in RAW264.7 macrophages treated with VPS34. The bacteria loads were determined at 1 h-post treatment by lysostaphin (50 μg/ml). USA300, *S. aureus* wild type; *moeA*, *moeA* mutant strain in USA300 background; *moeA*(p*moeA*), *moeA* mutant strain complement with pOS1-*moeA*.


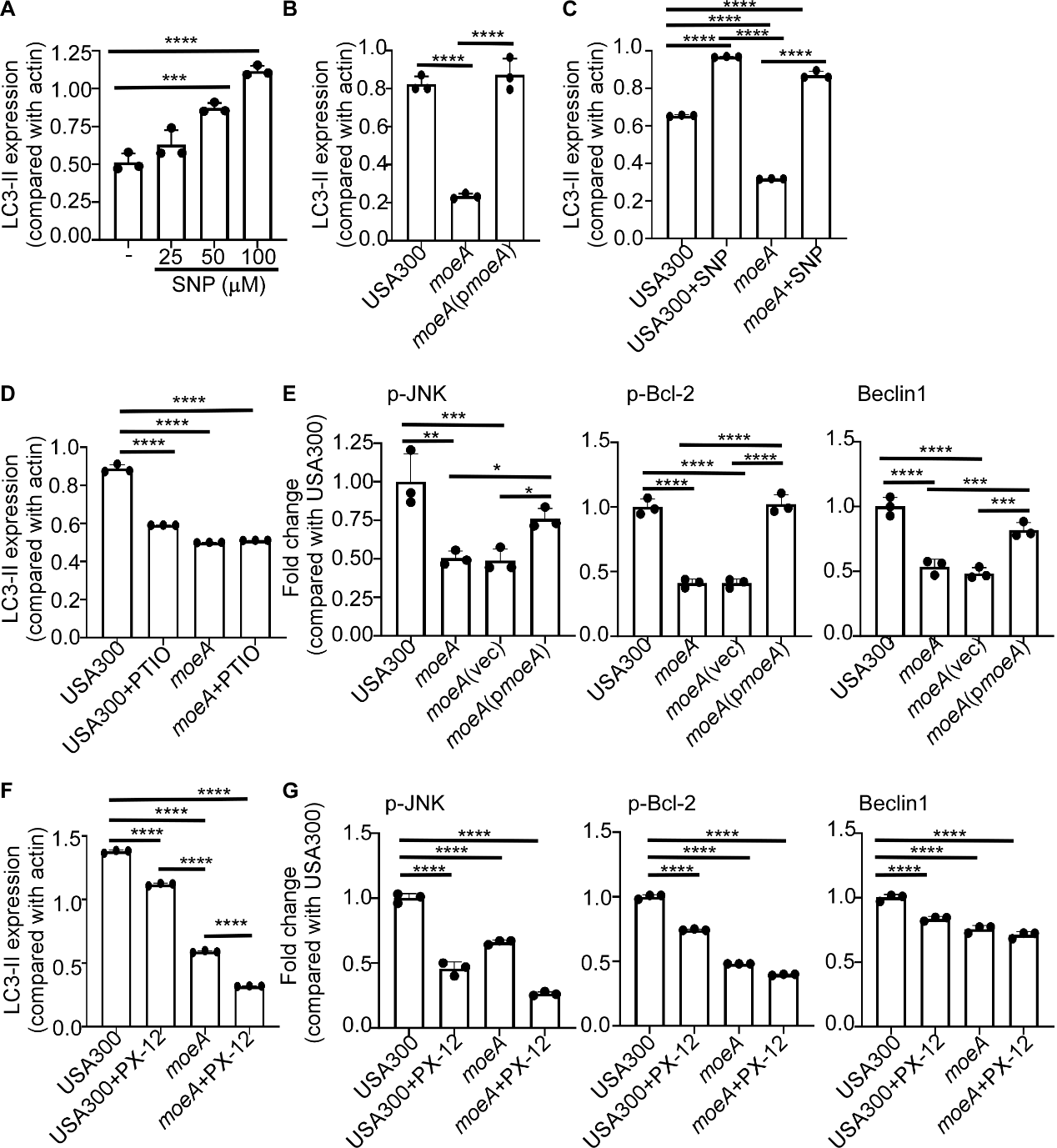


**FIG S3. The qualification for the Western blot. (A)** LC3-II for **Figure** 5A, **(B)** LC3-II for **Figure** 5B, **(C)** LC3-II for **Figure** 5C, **(D)** LC3-II for **Figure** 5F, **(E)** the phosphorylation of PNK (p-PNK) and Bcl-2 (p-Bcl-2), Beclin1 for **Figure** 5H, **(F)** LC3-II for **Figure** 5K and **(G)** the phosphorylation of PNK (p-PNK) and Bcl-2 (p-Bcl-2), Beclin1 for **Figure** 5L. The statistical significance was measured by one-way ANOVA. *, *P*  < 0.05; **, *P*  < 0.01; ***, *P*  < 0.001; ****, *P*  < 0.0001.


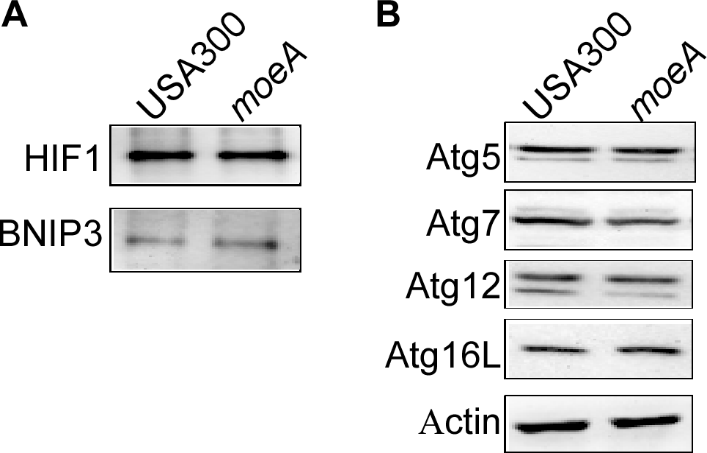


**FIG S4. The expression of autophagy-related proteins was compared in macrophages infected with different *S. aureus* strains.** RAW264.7 was infected with *S. aureus* (MOI=10) for 1 h, and then treated with lysostaphin (50 μg/ml) for 1 h. Total protein was extracted by RIPA (Radio [Immunoprecipitation](https://www.sciencedirect.com/topics/medicine-and-dentistry/immunoprecipitation) Assay) lysis buffer and separated by SDS-PAGE and transferred to PVDF membranes. After blocking and incubating with antibodies, observed protein expression level through HRP chemiluminescence method. USA300, *S. aureus* wild type; *moeA*, *moeA* mutant strain in USA300 background.


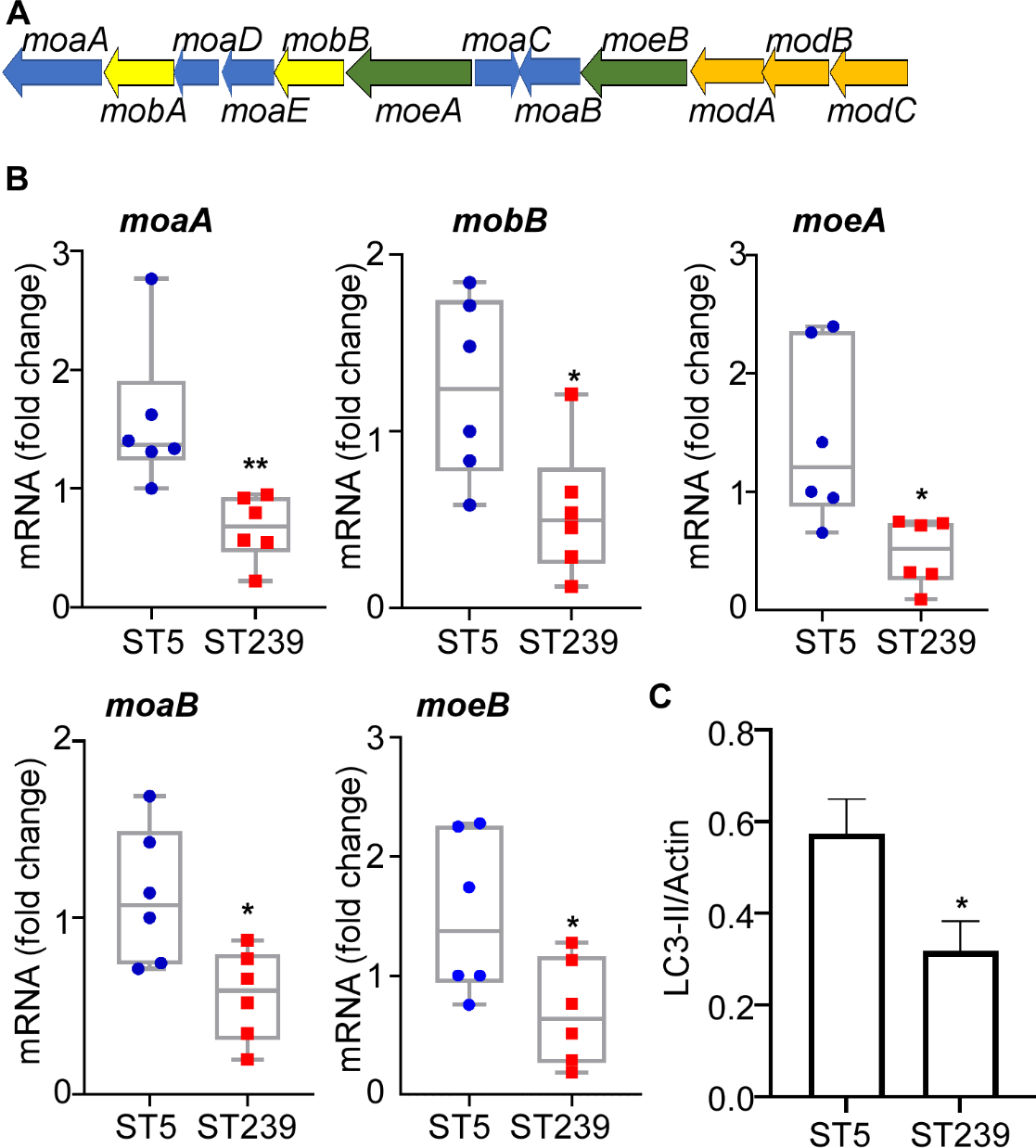


**FIG S5. The genes involved in the biosynthesis of molybdenum cofactor were highly expressed in *S. aureus* ST5. (A)** The gene operon involving in the biosynthesis of molybdenum cofactor. **(B)** The transcription levels of genes associated with the biosynthesis of molybdenum cofactor were compared between *S. aureus* ST5 and ST239. **(C)** The qualification of autophagy-related proteins in macrophages infected with different *S. aureus* strains. The data were collected from 2 biological repeats. The statistical significance was measured by two-tailed Student’s t-test. *, *P* < 0.05; **, *P* < 0.01.


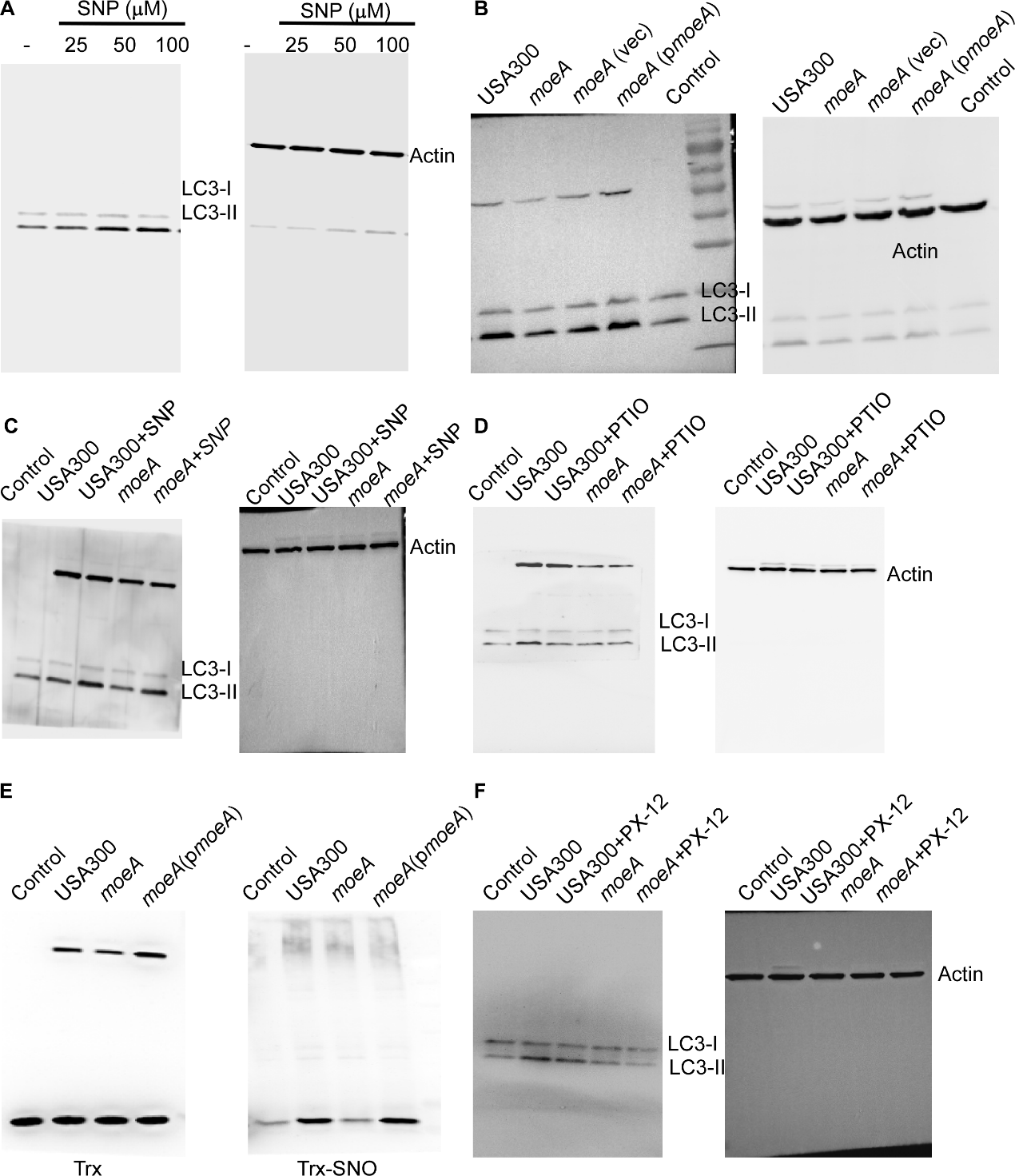


**FIG S6. The original gels for Western blot. (A)** for **Figure** 5A, **(B)** for **Figure** 5B, **(C)** for **Figure** 5C, **(D)** for **Figure** 5F, **(E)** for **Figure** 5G and **(F)** for **Figure** 5K.


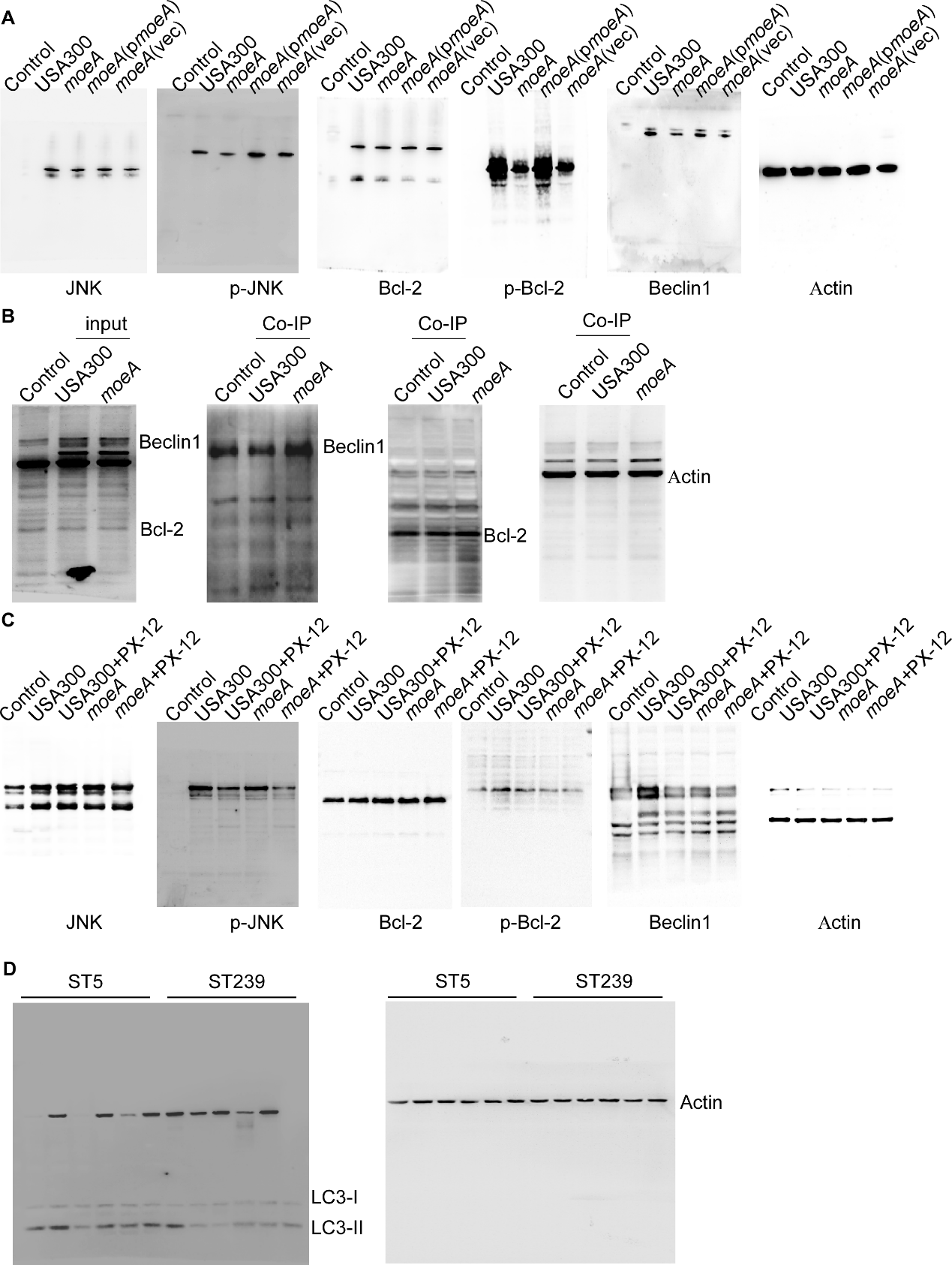


**FIG S7. The original gels for Western blot. (A)** for **Figure** 5H, **(B)** for **Figure** 5I, **(C)** for **Figure** 5L and **(D)** for **Figure** 8D.


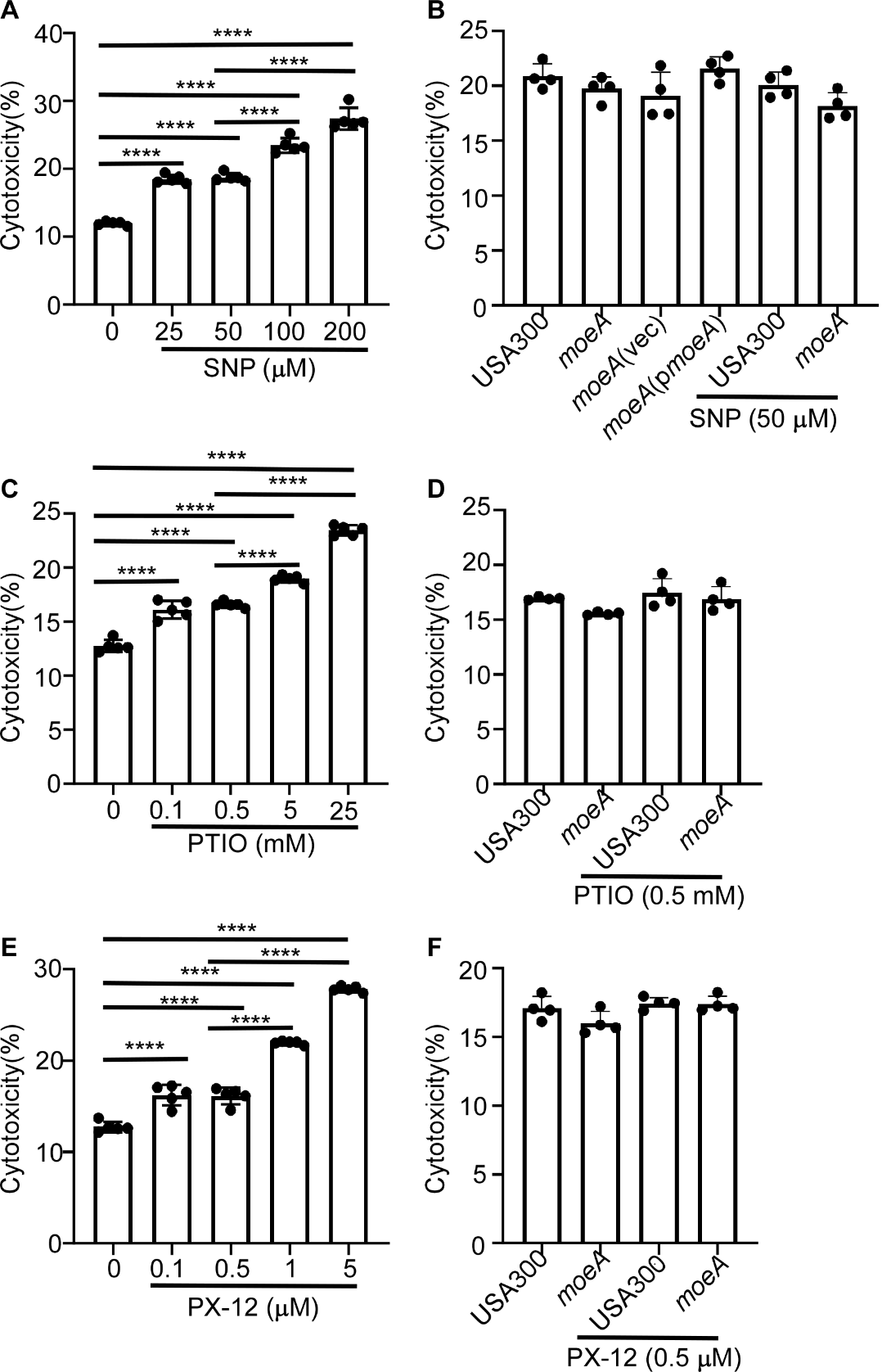


**FIG S8.** **The cytotoxic activity of macrophages.** The macrophages were treated with SNP **(A),** infected with different *S. aureus* strains when treated with 50 μM SNP **(B)**, carboxy-PTIO **(C)** , infected with different *S. aureus* strains when treated with 0.5 mM carboxy-PTIO **(D)**, PX-12 **(E),** or infected with different *S. aureus* strains when treated with 0.5 μM PX-12. The statistical significance was measured by one-way ANOVA. ****, *P*  < 0.0001.

**Table S1. The genes regulated significantly by RNA-seq between USA300 and the *moeA* deletion mutant.**

| **Gene name** | **Gene description** | **Log_2_FC*** | **Pvalue** | **Regulate** |
| --- | --- | --- | --- | --- |
| *SAUSA300_0793* | hypothetical protein | -1.29408 | 6.72E-05 | down |
| *SAUSA300_0795* | putative thioredoxin | -1.27447 | 8.01E-05 | down |
| *sspC* | I57 family staphostatin B | -0.92251 | 6.36E-05 | down |
| *sspB* | C47 family staphopain B | -1.05371 | 6.28E-06 | down |
| *sspA* | S01 family glutamyl endopeptidase | -1.25389 | 8.37E-05 | down |
| *moeA* | molybdopterin biosynthesis protein | -11.2993 | 7.01E-19 | down |
| *narK* | MFS family major facilitator transporter | 6.294518 | 2.7E-140 | up |
| *nreC* | transcriptional regulator | 1.426139 | 9.71E-06 | up |
| *nreB* | possible sensor histidine kinase | 1.448792 | 9.71E-05 | up |
| *nreA* | hypothetical protein | 1.343142 | 0.000372 | up |
| *narI* | nitrate reductase gamma subunit | 2.128306 | 5.36E-08 | up |
| *narJ* | nitrate reductase delta subunit | 5.958154 | 6.44E-28 | up |
| *narH* | nitrate reductase beta subunit | 6.205682 | 2.24E-31 | up |
| *narG* | nitrate reductase alpha subunit | 6.336249 | 1.89E-42 | up |
| *hemX* | uroporphyrin-III C-methyltransferase | 4.303899 | 1.63E-16 | up |
| *nirD* | nitrite reductase (NAD(P)H) small subunit | 4.928464 | 2.41E-14 | up |
| *nirB* | nitrite reductase (NAD(P)H) large subunit | 4.385468 | 3.46E-18 | up |
| *nirR* | transcriptional regulator NirR | 3.686954 | 5.84E-16 | up |
| *SAUSA300_2349* | FNT family formate-nitrite transporter | 0.987254 | 0.000158 | up |

***FC: fold change of gene expression (*moeA* / USA300)**

**Table S2. *S.aureus* clinical isolates used for the paper.**

| NO | Name | Resources | SpA typing | MLST |
| --- | --- | --- | --- | --- |
| 1 | 2015-2 | isolated from sputum | t214 | ST5 |
| 2 | 2015-58 | isolated from sputum | t311 | ST5 |
| 3 | 2016-216 | isolated from sputum | t311 | ST5 |
| 4 | 2016-0297 | isolated from sputum | t311 | ST5 |
| 5 | 2017-0669 | isolated from sputum | t2460 | ST5 |
| 6 | 2017-0913 | isolated from sputum | t2460 | ST5 |
| **7** | 2015-558 | isolated from sputum | t037 | ST239 |
| 8 | 2015-400 | isolated from sputum | t030 | ST239 |
| 9 | 2016-0907 | isolated from sputum | t030 | ST239 |
| 10 | 2016-0081 | isolated from sputum | t037 | ST239 |
| 11 | 2017-10 | isolated from sputum | t030 | ST239 |
| 12 | 2017-0764 | isolated from sputum | t632 | ST239 |

**Table S3. Oligonucleotides used in this study**

| **Name** | **Sequence (5’ 🡪 3’)** | **Target** |
| --- | --- | --- |
| *narK*-F | ACGTACGTGATTACTAGTGG | Real-time PCR for *narK* |
| *narK*-R | CTTAAGACCAATAGGTGGCAT | Real-time PCR for *narK* |
| *narI-*F | ATCGGCACACCACTCATCAATG | Real-time PCR for *narI* |
| *narI*-R | GCAGTGGGTGTGTCTGAGCAT | Real-time PCR for *narI* |
| *narJ*-F | AGTGCTTGAATCACATAGGC | Real-time PCR for *narJ* |
| *narJ*-R | GAACGTGGGCAAATGTTAGCT | Real-time PCR for *narJ* |
| *narH*-F | ATGGCTGTTGAGTAACTTG | Real-time PCR for *narH* |
| *narH*-R | GGTATTGGTTACGATTGGAT | Real-time PCR for *narH* |
| *narG*-F | ACGCAGTACCAAAGCATCTG | Real-time PCR for *narG* |
| *narG*-R | GGTCGACGATATTCACCAT | Real-time PCR for *narG* |
| *nreC*-F | ATGCGTCTTATGTGCTTC | Real-time PCR for *nreC* |
| *nreC*-R | GTTAGCTATTCGAACT | Real-time PCR for *nreC* |
| *nreB*-F | ACGCATCATACGTTGTTG | Real-time PCR for *nreB* |
| *nreB*-R | GCAATGAATGATGCTGCT | Real-time PCR for *nreB* |
| *nirD*-F | AGGTTCTTGAACAATACC | Real-time PCR for *nirD* |
| *nirD*-R | GTGACAACTATAGAT | Real-time PCR for *nirD* |
| *nirB*-F | AGTTGGTCTGCATCTG | Real-time PCR for *nirB* |
| *nirB*-R | CATCTAAAGAAGTTCGACAT | Real-time PCR for *nirB* |
| *nirR*-F | ATGCGTCACAAATACGTTG | Real-time PCR for *nirR* |
| *nirR*-R | CGACGGTATCCAGACAT | Real-time PCR for *nirR* |
| *moeA*-F | ATGGCGCAGATGCTGTTGT | Real-time PCR for *moeA* |
| *moeA*-R | ACCTCTGCATAGCCAT | Real-time PCR for *moeA* |
| *moaA*-F | GCGCGTGTACATGTAGAACA | Real-time PCR for *moaA* |
| *moaA*-R | AGAGCAGCACTTTGAAATCGA | Real-time PCR for *moaA* |
| *mobB*-F | CCGCTTCAAAATGCTTCATGT | Real-time PCR for *mobB* |
| *mobB* -R | AGTCACATGGTTATACAGTTGCT | Real-time PCR for *mobB* |
| *moaB*-F | GCGTCGTGATGGCTACTTTT | Real-time PCR for *moaB* |
| *moaB*-R | TGGGCGAACATCAAAACGTT | Real-time PCR for *moaB* |
| *moeB*-F | GTGCGTGTCATGCCATGTAT | Real-time PCR for *moeB* |
| *moeB* -R | ACGGAACAACCAATTGACAC | Real-time PCR for *moeB* |
| *gyrB*-F | CAAATGATCACAGCATTTGGTACAG | Real-time PCR for *gyrB* |
| *gyrB*-R | CGGCATCAGTCATAATGACGAT | Real-time PCR for *gyrB* |
| *iNOS*-F | CCAGGCTGGAAGCTGTAAC | Real-time PCR for *iNOS* |
| *iNOS*-R | AGTGATGGCCGACCTGAT | Real-time PCR for *iNOS* |
| *eNOS*-F | TTGAGGATGTGGCTGTGTG | Real-time PCR for *eNOS* |
| *eNOS*-R | GAGTTAGGCTGCCTGAGATG | Real-time PCR for *eNOS* |
| *gadph*-F | CTTAGCCCCCCTGGCCAAG | Real-time PCR for *GAPDH* |
| *gadph* -R | TGGTCATGAGCCCTTCCACA | Real-time PCR for *GAPDH* |
| *IL1β*-F | TGTAATGAAAGACGGCACACC | Real-time PCR for *IL1*β |
| *IL1β*-R | TCTTCTTTGGGTATTGCTTGG | Real-time PCR for *IL1*β |
| *IL6*-F | CTGCAAGAGACTTCCATCCAGTT | Real-time PCR for *IL6* |
| *IL6*-R | GGGAAGGCCGTGGTTGTC | Real-time PCR for *IL6* |
| *TNFa* -F | TCAAGGACTCAAATGGGCTTTC | Real-time PCR for *TNFa* |
| *TNFa*-R | TGCAGAACTCAGGAATGGACAT | Real-time PCR for *TNFa* |
| *map1lc3a*^-/-^-P1 | GAACCGCAGACGCATCTCT | For identification of *map1lc3a*^-/-^ mice |
| *map1lc3a*^-/-^-P2 | TGGGAGGCGTAGACCATGTA | For identification of *map1lc3a*^-/-^ mice |
| *map1lc3a*^-/-^-P3 | CCATCGGGGATCGAGGGA | For identification of *map1lc3a*^-/-^ mice |
| *map1lc3a*^-/-^-P4 | CACCGGATGATCTTGACCAACT | For identification of *map1lc3a*^-/-^ mice |
